# Toward precise CRISPR DNA fragment editing and predictable 3D genome engineering

**DOI:** 10.1093/jmcb/mjaa060

**Published:** 2020-10-30

**Authors:** Qiang Wu, Jia Shou

**Affiliations:** Center for Comparative Biomedicine, MOE Key Lab of Systems Biomedicine, State Key Laboratory of Oncogenes and Related Genes, Institute of Systems Biomedicine, School of Life Sciences and Biotechnology, Shanghai Jiao Tong University, Shanghai 200240, China

**Keywords:** CRISPR, DNA fragment editing, 3D genome engineering, repair mechanisms, chromatin loops, precise modifications, predictable indels

## Abstract

Ever since gene targeting or specific modification of genome sequences in mice was achieved in the early 1980s, the reverse genetic approach of precise editing of any genomic locus has greatly accelerated biomedical research and biotechnology development. In particular, the recent development of the CRISPR/Cas9 system has greatly expedited genetic dissection of 3D genomes. CRISPR gene-editing outcomes result from targeted genome cleavage by ectopic bacterial Cas9 nuclease followed by presumed random ligations via the host double-strand break repair machineries. Recent studies revealed, however, that the CRISPR genome-editing system is precise and predictable because of cohesive Cas9 cleavage of targeting DNA. Here, we synthesize the current understanding of CRISPR DNA fragment-editing mechanisms and recent progress in predictable outcomes from precise genetic engineering of 3D genomes. Specifically, we first briefly describe historical genetic studies leading to CRISPR and 3D genome engineering. We then summarize different types of chromosomal rearrangements by DNA fragment editing. Finally, we review significant progress from precise 1D gene editing toward predictable 3D genome engineering and synthetic biology. The exciting and rapid advances in this emerging field provide new opportunities and challenges to understand or digest 3D genomes.

## Introduction

The successful finishing of the Human Genome Project ushers in a new era to understand and engineer genomes by reverse genetics. However, the folding of 3-billion-bp 1D mammalian genomes, which are ∼2 m long, into 3D structures within cell nuclei of ∼5 µm in diameter adds another layer of complexity. The secret of 3D genome coding likely resides in the non-coding regions—the 97.5% of mammalian genomes—that were once assumed to be ‘junk DNA’ but are now regarded as ‘crown jewels’. Specifically, high-throughput mapping of functional genomic sequences has revealed numerous non-coding DNA elements, up to 8.4 million in number (Neph et al., 2012). In addition, junk DNA transcribes so-called ‘junk RNA’—numerous long non-coding RNA—whose functions are difficult to study (Cech and Steitz, 2014). The organizational and structural roles of these non-coding DNA elements in 3D genome regulation and function necessitate functional genetic experiments.

## Trekking across time: the long journey of reverse genetics leading to CRISPR and 3D genome editing

Genetic research focuses on heredity and ‘mutants’ (Castle and Little, 1909; Muller, 1930). Some mutants arise spontaneously but specific mutants are usually generated through tedious forward genetic screening experiments (Acevedo-Arozena et al., 2008). Forward genetic screening in mice was performed before the mouse genome sequencing was finished and greatly contributed to our understanding of human physiology (Kile and Hilton, 2005). However, reverse genetics that would generate specific alterations of mammalian genomic sequences or so-called gene targeting was a dream in the early days.

### Transgenic: random integration in animal and plant genomes

**Figure 1:**
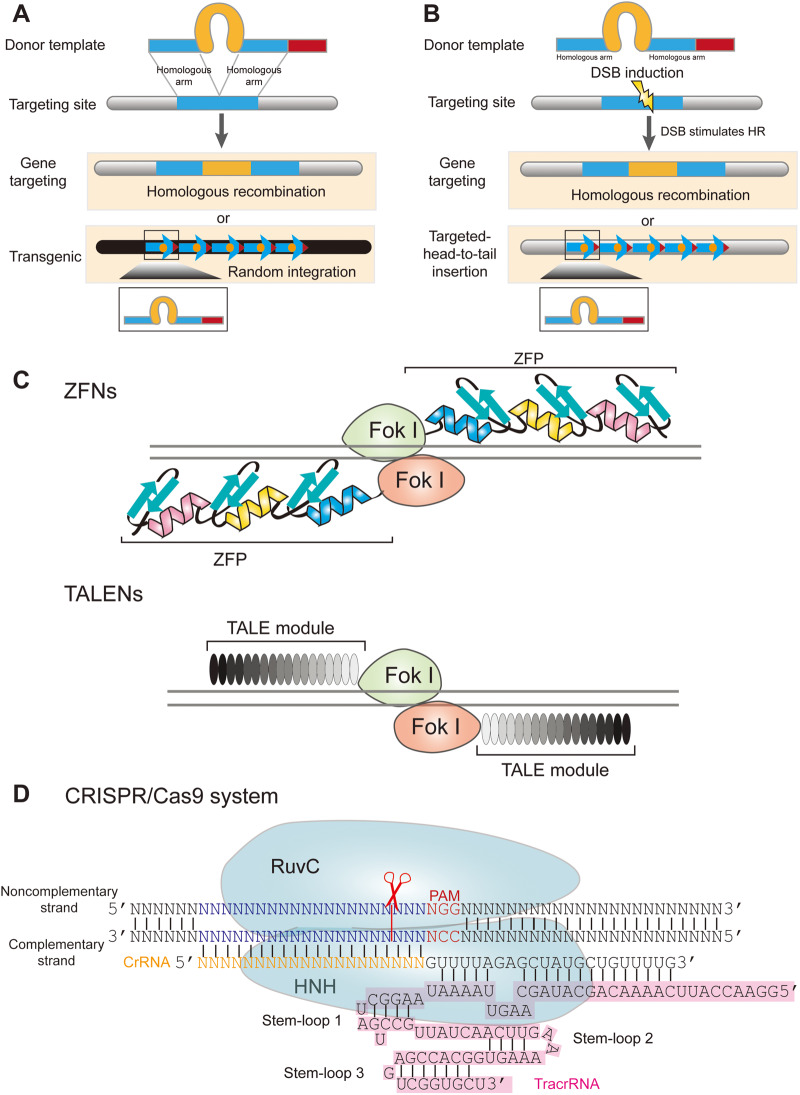
Schematic of genetic methods for specific genome modifications. (**A**) Gene targeting is achieved by sequence replacement with a donor template harboring designed sequences flanked by two homologous arms in a specific genome locus. In addition to targeted replacement, occasional random integration in a non-specific genome site results in transgenic insertion of a tandem concatemer. (**B**) DSB greatly stimulates gene targeting but not random transgenic integration. However, it can also result in targeted head-to-tail insertion at the DSB site. (**C**) A simplified illustration of gene editing by ZFNs and TALENs. In ZFNs, each zinc-finger recognizes three specific nucleotides. In TALENs, each nucleotide is recognized by a TALE repeat, which carries two specific amino acids. ZFP, zinc-finger protein. (**D**) The type II CRISPR/Cas9 system. Cas9 nuclease is programmed by CRISPR RNA (crRNA) and trans-activating CRISPR RNA (tracrRNA), which can be fused into a single synthetic guide RNA (sgRNA).

Transgenes were originally derived from viruses and transposons or so-called jumping genes in animals and plants (McClintock, 1950; Jaenisch and Mintz, 1974; Bevan et al., 1983). A transgene can be integrated randomly into one or very few sites of the mouse genome and exhibits expression patterns with position-effect variegations (Figure 1A; Jaenisch and Mintz, 1974; Gordon et al., 1980; Brinster et al., 1981; Costantini and Lacy, 1981). Multiple copies of transgenes are typically integrated at a random genomic site in tandem arrays as a head-to-tail concatemer (Figure 1A; Brinster et al., 1981; Folger et al., 1982). Homologous recombination (HR) was demonstrated convincingly to be the predominant mechanism of head-to-tail transgene integration (Folger et al., 1982). In fact, it is with this conviction that eventually led to the development of gene targeting in mice (Capecchi, 2005).

### Gene targeting or knockout mice

Gene targeting is different from transgenic technologies and has greatly accelerated biological researches. Even before the completion of the mouse genome sequencing, the dream of specific modification of any mouse locus had been realized by so-called gene targeting (Figure 1A; Smithies et al., 1985; Thomas et al., 1986). The technique is achieved by constructing a targeting vector with designed modification in a specific locus, which is flanked by two homologous arms. This donor template is then introduced into mouse embryonic stem cells (ESCs) (Evans and Kaufman, 1981; Martin, 1981) and replaces the endogenous sequences through HR (Figure 1A). Finally, the ESC clones carrying the designed specific modification are then injected into the mouse blastocoel cavity to generate chimeric mice. Heterozygous or homozygous mice could then be obtained simply by breeding. The remarkable technique and general protocol for generating knockout mice with any gene targeted were quickly developed (Mansour et al., 1988).

### Gene editing with zinc-finger nucleases, transcription activator-like effector nucleases, and CRISPR

Targeted gene replacement through HR has also been achieved for other model organisms such as yeast and flies (Scherer and Davis, 1979; Rong and Golic, 2000). Since free double-strand break (DSB) ends greatly stimulate HR (Figure 1B; Orr-Weaver et al., 1981; Jasin and Berg, 1988), intense efforts were devoted to creating targeted DSBs. A series of programmable endonucleases, including zinc-finger nucleases (ZFNs) (Bibikova et al., 2003), transcription activator-like effector nucleases (TALENs) (Miller et al., 2011), and clustered regularly interspaced short palindromic repeat/CRISPR-associated nuclease 9 (CRISPR/Cas9) (Gasiunas et al., 2012; Jinek et al., 2012; Cong et al., 2013; Mali et al., 2013), were found to be able to introduce not only targeted modifications across genomes but also targeted head-to-tail insertions (Figure 1B‒D; Folger et al., 1982; Skryabin et al., 2020). CRISPR, in particular, has revolutionized targeted genome modification because of its simplicity and practicality.

### CRISPR: clustered regularly interspaced short palindromic repeats

CRISPR/Cas9 is an RNA-guided adaptive immune system of bacteria and archaea, which defends against phage or virus infection and plasmid conjugation. The type II CRISPR/Cas9 system has been widely used for genome editing. The programmable CRISPR/Cas9 system consists of a synthetic single-guide RNA (sgRNA; derived from crRNA and tracrRNA) and RNA-guided Cas9 nuclease (Figure 1D; Jinek et al., 2012). Upon recognition of a protospacer adjacent motif (PAM, NGG for *Sp*Cas9 from *Streptococcus pyogenes*) downstream of the targeting sequence, Cas9 cleaves the complementary and non-complementary strands of the target DNA duplex by the HNH and RuvC nuclease domains, respectively (Garneau et al., 2010; Gasiunas et al., 2012; Jinek et al., 2012), resulting in presumed blunt-ended DSBs which are then ligated by cellular endogenous DNA repair machineries (Figure 1D).

### Gene-editing outcomes from single DSBs

There are numerous gene-editing applications of single DSBs from CRISPR. The simplest application is the generation of frameshift mutations in the coding region of a protein-encoding gene. Cas9 can be reprogrammed by single sgRNAs to target a coding exon, generating one DSB that often leads to nucleotide insertions and/or deletions (indels). Two-thirds of these indels can cause a shift in the open reading frame of a protein-coding gene, resulting in truncated protein translation or null mutation through the nonsense-mediated mRNA decay. Recent studies demonstrated, however, that single DSBs also lead to large deletions from extended long resections (Li et al., 2015a; Shin et al., 2017; Kosicki et al., 2018, 2020; Jia et al., 2020). In addition, Cas9 with single sgRNAs causes frequent loss-of-heterozygosity or gene conversion as well as allele-specific chromosomal removal in human embryos (Alanis-Lobato et al., 2020; Liang et al., 2020; Zuccaro et al., 2020). Finally, if a donor DNA template is provided, single DSBs often lead to targeted precise gene insertions through HR (Figure 1B).

### 3D genome primer

Although genetic information is encoded in the finished linear 1D genomic sequences, the extremely long and thin DNA molecules do actually exist in Euclidean 3D space and are physically folded into a cell nucleus. Each interphase chromosome occupies a distinct territory and compartmentalizes further into multiple topologically associated domains (TADs). The recognition sites of architectural protein CCCTC-binding factor (CTCF) are enriched at boundaries of chromatin domains; however, there are also numerous CTCF sites located within topological domains or TADs. Exactly how 3D genomes are folded and regulated remains unknown; however, novel technological developments have enabled tremendous progress in 3D genomics (Banigan and Mirny, 2020; Li et al., 2020a; Zhang and Li, 2020). In particular, DNA fragment editing or CRISPR-induced chromosomal rearrangements have shed significant insights into the mechanisms of 3D genome folding (Liu and Wu, 2020).

There are numerous excellent reviews on CRISPR or 3D genomics (Doudna and Charpentier, 2014; Huang and Wu, 2016; Jiao and Gao, 2016; Yan and Li, 2019; Yang and Huang, 2019; Zhang, 2019; Anzalone et al., 2020; Li et al., 2020a; Yang and Chen, 2020; Zhang and Li, 2020; Zhang et al., 2020). Here, we focus on chromosomal rearrangements and 3D genome engineering by DNA fragment editing using Cas9 with dual sgRNAs.

## Chromosomal rearrangements by CRISPR with dual sgRNAs

**Figure 2:**
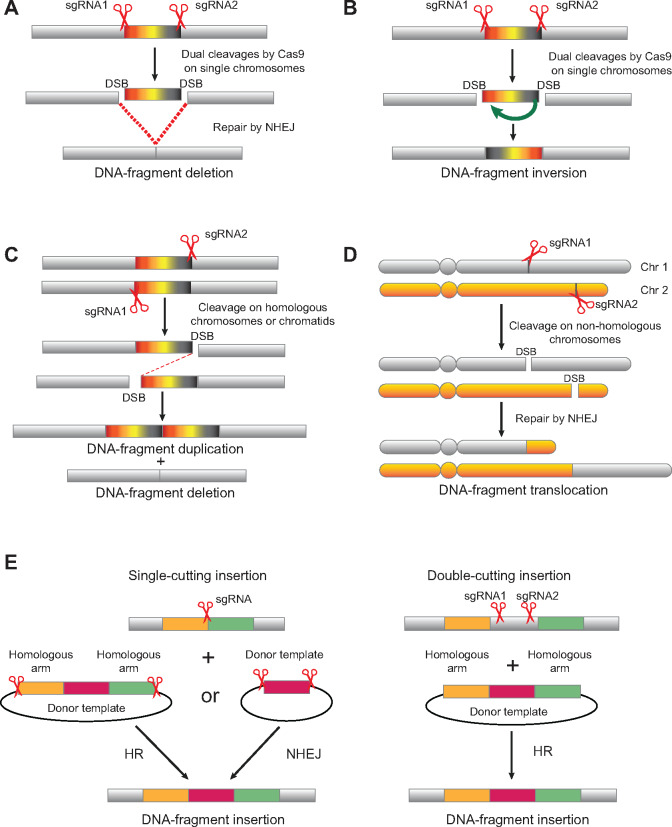
DNA fragment editing induces chromosomal rearrangements including large DNA fragment deletion (**A**), inversion (**B**), duplication (**C**), translocation (**D**), as well as insertion (**E**).

Structural chromosomal abnormalities or chromosomal rearrangements include DNA fragment deletions, inversions, duplications, translocations, and insertions (Figure 2; Shaffer and Lupski, 2000; Huang and Wu, 2016). Chromosomal rearrangements are estimated to occur at 0.6% of human newborns (Jacobs et al., 1992). In addition, recurrent chromosomal rearrangements are quite frequent in human neurological diseases (Weckselblatt and Rudd, 2015) and tumors (Rabbitts, 1994; Mitelman et al., 1997). Early studies to model human diseases generated large chromosomal rearrangements of up to tens of millions bp in mice through the combined technologies of gene targeting and Cre/*LoxP* recombination (Ramirez-Solis et al., 1995; Herault et al., 1998; Wu et al., 2007; and reviewed in Mills and Bradley, 2001; Yu and Bradley, 2001). ZFNs and TALENs have also been used to generate chromosomal rearrangements in human cells (Lee et al., 2010; Gupta et al., 2013; Nyquist et al., 2013; Xiao et al., 2013). In this section, we outline 3D genome engineering by modeling chromosomal rearrangements using the CRISPR/Cas9 system with dual sgRNAs (Figure 2; Li et al., 2015b).

### Chromosomal rearrangements by DNA fragment editing

Disruption of a specific gene of interest could be easily achieved by Cas9 reprogrammed with single sgRNAs because two-thirds of random indels at a DSB site within a protein-coding region result in frameshifts. For non-coding elements, however, random indels induced by Cas9 with single sgRNAs are usually not enough. A practical way to characterize non-coding regions, of which there are estimated millions in mammalian genomes, is to generate very large deletions containing defined regions with multiple non-coding elements (Wu et al., 2007). Engineering a large DNA fragment could be achieved by Cas9 reprogrammed with dual sgRNAs, which would generate two concurrent DSBs in a genome (Figure 2). Specifically, with the participation of cellular DNA repair proteins, the four DSB ends generated by the two Cas9 cleavages are randomly ligated, resulting in DNA fragment deletion or inversion when concurrent DSBs occur on single chromosomes (Figure 2A and B) and DNA fragment duplication or translocation when the DSBs occur on different chromatids or chromosomes (Figure 2C and D).

### DNA fragment deletion by CRISPR

It is well established that Cas9 with dual sgRNAs can easily generate DNA fragment deletions (Figure 2A; Huang and Wu, 2016). However, initial utilization of the CRISPR system with dual sgRNAs has been to mitigate off-target activity. The D10A Cas9 nickase guided by paired sgRNAs in proper configurations and optimized offsets generates double nicking and 5′ overhangs (Ran et al., 2013; Shen et al., 2014). Subsequent targeting of two separate intrachromosomal sites by wildtype Cas9 with dual sgRNAs results in the interstitial deletion of large DNA fragments in zebrafish (Gupta et al., 2013; Xiao et al., 2013), mammalian cells (Cong et al., 2013; Mali et al., 2013; Canver et al., 2014; Guo et al., 2015, 2018; He et al., 2015; Li et al., 2015c; Kim et al., 2017; Schmieder et al., 2018; Shou et al., 2018; Shi et al., 2019; Jia et al. 2020), mice (Zhou et al., 2014; Li et al., 2015a; Jia et al., 2020), rabbits (Song et al., 2016), worms (Chen et al., 2014), and plants (Pauwels et al., 2018; Schmidt et al., 2019) (Table 1).

**Table 1:** Chromosomal rearrangements by CRISPR with dual sgRNAs.

Chromosomal rearrangement	Cell type or organism	Gene or region of interest	Targeting size (kb)	Targeting efficiency (%)	Efficiency measuring method	References
DNA fragment deletion	Mice	Hypoxanthine phosphoribosyltransferase locus (*HPRT*)	10	9/27 (33.3%)	Mutant mice by zygote injection	Fujii et al. (2013)
	Murine erythroleukemia (MEL) cells	ND	1.3	18/48 (37.5%)	Screening single cell clones	Canver et al. (2014)
			2.0	60/234 (25.6%)		
			2.8	29/78 (37.2%)		
			4.5	14/122 (11.5%)		
			4.5	10/164 (6.1%)		
			7.3	59/332 (17.8%)		
			8.0	190/800 (23.8%)		
			13.5	20/160 (12.5%)		
			15.0	74/316 (23.4%)		
			19.0	2/68 (2.9%)		
			19.0	21/240 (8.8%)		
			20.3	34/140 (24.3%)		
			23.0	20/142 (14.1%)		
			23.0	5/54 (9.3%)		
			70.5	1/364 (0.3%)		
			1025.3	1/266 (0.4%)		
			1025.7	3/420 (0.7%)		
	HAP1 cells	Chr 15: 61,105,000 to ~89,890,000	~28000	5/400 (1.3%)	Screening single cell clones	Essletzbichler et al. (2014)

	Mouse ESC	*Dip2a*	65	11/93 (11.8%)	Screening single cell clones	Zhang et al. (2015)
	Mice	*Dip2a*	65	3/14 (21.4%)	Mutant mice by zygote injection	Zhang et al. (2015)
	Mice	*Rab38*	3.2	10/27 (37%)	Mutant mice by zygote injection	Brandl et al. (2015)
	HEK293FT cells	*HPRT*	1.79	3.3%	Digital PCR analysis	He et al. (2015)
			2.14	3.3%		
			13.33	10%		
			0.35	10%		
			11.54	10%		
			11.19	1%		
			63.07	10%		
			112.93	10%		
			513.60	10%		
			1017.84	1%		
	HEK293FT cells	Hypoxanthine phosphoribosyltransferase locus (*HPRT*)	513.60	8/63 (12.7%)	Screening single cell clones	He et al. (2015)
	Mouse ESC	*H2afy*	1.189	11/288 (3.8%)	Screening single cell clones	Kraft et al. (2015)
		*Bmp2*	3.7	12/192 (6.3%)		
		*Ihh*	12.6	121/288 (42%)		
		*Pitx1*	32	9/288 (3.1%)		
		*Laf4*	353	38/288 (13.2%)		
		*Epha4*	1672	4/192 (2.1%)		
	HEK293T	*β-globin RE1*	0.709	(28.33 ± 6.19)%	Quantitative PCR	Li et al. (2015a)
		*Pcdh RE1*	1.272	(17.51 ± 1.04)%		
		*β-globin RE2*	6.277	(34.49 ± 3.57)%		
		*HoxD*	18.142	(9.15 ± 0.11)%		
		*β-globin*	80.732	(13.39 ± 0.80)%		
		*Pcdhα cluster*	256.744	(8.46 ± 0.24)%		
		*Pcdh α, β, and γ clusters*	807.480	(0.47 ± 0.08)%		
	Mice	*Pcdh* locus 1	1.241	26/120 (21.7%)	Mutant mice by zygote injection	Li et al. (2015a)
		*Pcdh* locus 2	0.96	6/8 (75%)		
		*Pcdh* locus 3	29.401	5/26 (19.2%)		
	Mice	Tyrosinase (*Tyr*) non-coding regulatory DNA elements	1.2	19/64 (29.7%)	Mutant mice by zygote injection	Seruggia et al. (2015)
	Human Pluripotent Stem Cells (hESC)	*MALAT1*	0.5	7/12 (58.3%)	Screening single cell clones	Liu et al. (2016)
			1	6/8 (75%)		
			3	18/32 (56.3%)		
			8	18/39 (46.2%)		
	Mice	*Tyr*	9.5	3/30 (10%)	Mutant mice by zygote injection	Boroviak et al. (2016)
		*Tyr*	65	13/81 (16%)		
		*Nox4*	155	11/46 (23.9%)		
		*Grm5*	545	12/68 (17.6%)		
		*Nox4* to *Grm5*	1150	14/48 (29.2%)		
	Rats	*Cbs*	37.2	12/24 (50%)	Mutant rat by zygote injection	Birling et al. (2017)
		*Dyrk1a*	121.7	4/28 (14.3%)		
		*Umodl1-Prmt2*	3513	2/40 (5%)		
		*Lipi-Zfp295*	24499	1/9 (11.1%)		
	Mice	*Hmgn1*	16.8	4/8 (50%)	Mutant mice by zygote injection	Birling et al. (2017)
		*Tiam1*	226	8/41 (19.5%)		
		*Runx1-Cbr1*	1100	1/34 (2.9%)		
	CHO cells (Chinese Hamster Ovary cells)	α-1,6-Fucosyltransferase 8 (*FUT8*)	2.1	34%	Quantitative PCR	Schmieder et al. (2018)
			12.5	30%		
			52.6	29%		
			96.8	35%		
			150.7	21%		
	Rabbits	Tyrosinase (*Tyr*)	105	3/17 (17.6%)	Mutant rabbits by zygote injection	Song et al. (2016)
	Rabbits	*GJA8*	0.054	11/11 (100%)	Mutant rabbits by zygote injection	Yuan et al. (2016)
	Pigs	*PDX1*	0.204	3/9 (33.3%)	Mutant pigs by zygote injection	Wu et al. (2017)
	Rhesus monkeys	*PINK1*	7.237	3/11 (27.3%)	Mutant monkeys by zygote injection	Yang et al. (2019)
DNA fragment inversion	HEK293T	*KIF5B–RET*	11000	1.6%	Flow cytometry	Choi and Meyerson (2014)
		*EML4–ALK*	12000	8%	Flow cytometry	
	Mice	*EML4–ALK*	11000	1.5 × 10^−^^6^	PCR	Blasco et al. (2014); Maddalo et al. (2014)
	Patient iPSCs	*F8 gene*	140	8/120 (6.7%)	Screening single cell clones	Park et al. (2015)
			563	5/135 (3.7%)		
	Murine erythroleukemia (MEL) cells	ND	2	20/156 (12.8%)	Screening single cell clones	Canver et al. (2014)
			8	9/96 (9.4%)		
			15	17/164 (10.4%)		
			20.3	26/140 (18.6%)		
			1025.3	2/266 (0.8%)		
			1025.7	2/418 (0.5%)		
	Mouse ESC	*H2afy*	1.189	2/288 (0.7%)	Mutant mice by zygote injection	Kraft et al. (2015)
		*Bmp2*	3.7	3/192 (1.6%)		
		*Ihh*	12.6	7/288 (2.4%)		
		*Pitx1*	32	3/288 (1%)		
		*Laf4*	353	12/288 (4.2%)		
		*Epha4*	1672	3/192 (1.6%)		
	HEK293T	*β-globin RE1*	0.709	(21.12 ± 4.99)%	Quantitative PCR	Li et al. (2015a)
		*Pcdh RE1*	1.272	(23.28 ± 2.42)%		
		*β-globin RE2*	6.277	(23.13 ± 1.13)%		
		*HoxD*	18.142	(7.28 ± 1.60)%		
		*β-globin*	80.732	(5.96 ± 0.28)%		
		*Pcdhα cluster*	256.744	(5.48 ± 0.37)%		
		*Pcdh α, β, and γ clusters*	807.480	(0.71 ± 0.12)%		
	Mice	*Pcdh* locus 1	1.241	6/120 (5%)	Mutant mice by zygote injection	Li et al. (2015a)
		*Pcdh* locus 2	0.96	8/8 (100%)		
		*Pcdh* locus 3	29.401	2/26 (7.7%)		
	Mice	Tyrosinase (*Tyr*) non-coding regulatory DNA elements	1.2	7/64 (10.9%)	Mutant mice by zygote injection	Seruggia et al. (2015)
	Mice	*Nox4*	155	14/46 (30.4%)	Mutant mice by zygote injection	Boroviak et al. (2016)
		*Grm5*	545	12/68 (17.6%)		
		*Nox4* to *Grm5*	1150	10/48 (20.8%)		
	Rat	*Cbs*	37.2	7/24 (29.2%)	Mutant rat by zygote injection	Birling et al. (2017)
		*Dyrk1a*	121.7	3/28 (10.7%)		
	Mice	*Runx1-Cbr1*	1100	1/34 (2.9%)	Mutant mice by zygote injection	Birling et al. (2017)
DNA fragment duplication	Mouse ESC	*Pitx1*	32	2/288 (0.7%)	Screening single cell clones	Kraft et al. (2015)
		*Laf4*	353	81/288 (28.1%)		
	HEK293T	*Pcdh RE1*	1.272	(0.23 ± 0.12)%	Quantitative PCR	Li et al. (2015a)
		*β-globin RE2*	6.277	(5.30 ± 1.19)%		
		*β-globin*	80.732	(5.97 ± 0.33)%		
		*Pcdhα cluster*	256.744	(0.61 ± 0.02)%		
		*Pcdh α, β, and γ clusters*	807.480	(0.17 ± 0.03)%		
	Mice	*Pcdh* locus 1	1.241	1/26 (3.8%)	Mutant mice by zygote injection	Li et al. (2015a)
	Mice	*Nox4*	155	1/46 (2.2%)	Mutant mice by zygote injection	Boroviak et al. (2016)
		*Grm5*	545	1/68 (1.5%)		
	Rat	*Cbs*	37.2	1/24 (4.2%)	Mutant rat by zygote injection	Birling et al. (2017)
		*Dyrk1a*	121.7	2/28 (7.1%)		
		*Lipi-Zfp295*	24499	1/9 (11.1%)		
	Mice	*Tiam1*	226	1/41 (2.4%)	Mutant mice by zygote injection	Birling et al. (2017)

### DNA fragment inversion by CRISPR

In addition to DNA fragment deletions, DNA fragment inversion events also occur through double cutting, which is different from double nicking, within single chromosomes (Figure 2B). Different from DNA fragment deletion, in which there is only one junction after deleting the intervening sequences, DNA fragment inversion has an upstream junction and a downstream junction after inverting the intervening DNA fragment (Huang and Wu, 2016).

DNA fragment inversions using Cas9 guided with dual sgRNAs can be easily achieved in cultured cells (Canver et al., 2014; Choi and Meyerson, 2014; Guo et al., 2015; Kraft et al., 2015; Li et al., 2015a; Park et al., 2015), mice (Blasco et al., 2014; Maddalo et al., 2014; Kraft et al., 2015; Li et al., 2015a; Seruggia et al., 2015; Boroviak et al., 2016; Birling et al., 2017; Lu et al., 2019; Jia et al. 2020), rats (Birling et al., 2017), and plants (Schmidt et al., 2019). In particular, DNA fragment inversion results in the generation of an oncogenic gene from fusion of two genes at an inversion junction in mouse somatic tissues that faithfully models human tumors (Blasco et al., 2014; Maddalo et al., 2014). Finally, Cas9 guided by dual sgRNAs has been used to study the role of the orientation of non-coding regulatory elements such as enhancers and insulators (Guo et al., 2015; Li et al., 2015a).

### DNA fragment duplication by CRISPR

Chromosomal duplications can be generated by *trans*-allelic ligations of DSB ends in two homologous chromosomes or chromatids (Figure 2C; Golic and Golic, 1996; Wu et al., 2007; Li et al., 2015a). Specifically, DNA fragment duplications can be generated by complementary *trans*-chromatid ligations of paracentric DSB ends resulting from cleavages by Cas9 guided with dual sgRNAs after DNA replication during both mitosis and meiosis. Thus, Cas9 guided with dual sgRNAs induces DNA fragment duplications in cultured cells (Kraft et al., 2015; Li et al., 2015a). In addition, DNA fragment duplications in mice *in vivo* can be induced by Cas9 with dual sgRNAs through pronuclear microinjection (Li et al., 2015a; Korablev et al., 2017). In particular, a tandem duplication of a 1211-bp DNA fragment was confirmed by Sanger sequencing of the entire duplicated segment (Li et al., 2015a). Finally, quantitative analyses revealed frequent segmental duplications by Cas9 with dual sgRNAs, though with lower efficiency compared with that of DNA fragment deletions and inversions (Li et al., 2015a).

### Chromosomal translocation by CRISPR

Chromosomal translocations result from joining DSB ends in two distinct chromosomes (Figure 2D). Recurrent chromosomal translocations are frequent in many types of tumors especially in leukemias (Lieber, 2016; Vanoli and Jasin, 2017; Brunet and Jasin, 2018; Cheong et al., 2018). Cas9 reprogrammed with dual sgRNAs that target specific loci in non-homologous chromosomes has been used to induce chromosomal translocations to model human Ewing’s sarcoma, desmoplastic small round cell tumors, and acute myeloid leukemia (AML) (Torres et al., 2014; Vanoli et al., 2017).

### Relationship between DNA fragment size and editing frequency

Deletion frequencies at some loci are inversely correlated with the sizes of the intervening sequences between the two cleavage sites (Canver et al., 2014). However, at other loci, there is no inverse correlation between DNA-fragment-deletion frequency and the fragment size (Table 1; He et al., 2015; Kraft et al., 2015; Li et al., 2015a; Schmieder et al., 2018). In addition, the frequencies of DNA-fragment inversion and DNA-fragment duplication have no relationship with fragment sizes (Table 1). The DNA fragment-editing frequency may be related to the locus-specific 3D chromatin structure as well as the spatial distance between the two cutting sites, which is an unresolved problem requiring further studies.

### DNA fragment insertion by CRISPR

DNA fragment insertion can be efficiently achieved through the CRISPR system using Cas9 with either dual sgRNAs or single sgRNAs (Figure 2E). Mechanistically, DNA fragment insertions can be achieved by either HR or non-homologous end-joining (Suzuki et al., 2016). It is known that single cuts by Cas9 stimulate DNA fragment insertion through HR with a donor template harboring flanking homologous arms. One study carefully investigated the DNA fragment insertion efficiencies of HR by Cas9 with dual sgRNAs (Byrne et al., 2015). Moreover, Cas9 with dual sgRNAs targeting both the genome and donor template may be more efficient through homology-mediated end joining (HMEJ) (Yao et al., 2017). However, insertion needs careful screening for single-copy insertional clones or mice because any donor template could result in random head-to-tail tandem insertions just as transgenes (Figure 1B; Folger et al., 1982; Skryabin et al., 2020). Thus, the DNA fragment insertion clones or mice are best screened by Southern blot analyses rather than by PCR only.

## Many ways to cut and heal

The mutated sequences obtained from CRISPR/Cas9-editing result from eventual consequences of the opposite forces of Cas9 cleavage and cellular repair. Specifically, the observed random indels by Cas9 with single sgRNAs are eventual repaired outcomes after cycles of repeated ligation and cleavage of precisely ligated DNA ends. In addition to blunt-end cleavage, Cas9 can also cohesively cleave the DNA duplex generating staggered ends with 5′ overhangs. Thus, the cohesive cleavage of Cas9 actually generates diverse profiles of DSB ends with distinct 5′ overhangs. Finally, rapid progress in the field has made it possible to predict editing outcomes by manipulating DNA repair pathways (Long, 2019; Yeh et al., 2019).

### Double cutting vs. single cutting

The plain difference between cleavages of double and single cutting is that double cutting generates four DSB ends. The combinatorial ligations of two of these four DSB ends result in a variety of chromosomal rearrangements (Figure 2). The fundamental difference between double and single cutting is that in single cutting, after precise ligation of the two DSB ends, the repaired sequences still match the targeting sgRNA and thus can be recut. In contrast, the ligations of combinatorial two DSB ends out of the four ends from double cutting cannot be recut since the rearranged junctional sequences no longer match either of the two targeting sgRNAs (Huang and Wu, 2016; Shou et al., 2018; Shi et al., 2019). Therefore, dual-sgRNA-mediated chromosomal rearrangements maintain the integrity of Cas9-cleavage ends and make them less vulnerable to end-processing by repair enzymes (Figure 2). Hence, precise ligations upon direct rejoining of Cas9 blunt-cleavage ends after double cutting are much more frequent than after single cutting (Li et al., 2015a; Zhu et al., 2016b; Guo et al., 2018; Shou et al., 2018).

### Cohesive Cas9 cleavage in vitro and in silico

**Figure 3:**
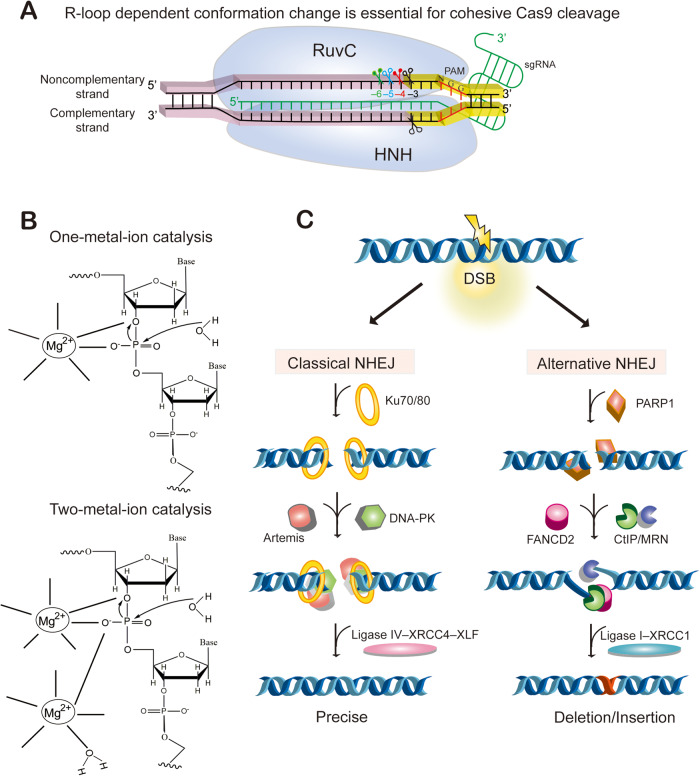
Mechanisms of cohesive Cas9 cleavage and repair. (**A**) Cas9 endonuclease reprogrammed by a synthetic guide RNA (sgRNA) can target any specific site in a genome through forming a structure composed of three strands of nuclear acid chains, known as R-loop. Specifically, the first 20 nucleotides of the sgRNA form a DNA‒RNA hybrid with 20 nucleotides of the targeting DNA sequences through base-pairing with the complementary strand, displacing the non-complementary strand (the original protospacer sequences) and resulting in a structure called R-loop. SgRNA guides Cas9 to the targeting site and Cas9 cleaves dsDNA at locations upstream of the PAM site. While the HNH domain of Cas9 cleaves the complementary strand at the exact −3 position upstream of the PAM site, the RuvC domain of Cas9 flexibly cleaves the non-complementary strand at the −6, −5, and −4 positions as well as the −3 position upstream of the PAM site, generating a diverse cohesive DSB ends with 1-, 2-, and 3-nt 5′ overhangs in addition to blunt ends. (**B**) Diagram of one-metal-ion cleavage mechanism for HNH and two-metal-ion cleavage mechanism for RuvC domain of Cas9 protein. (**C**) Schematic of NHEJ repair pathways for repairing of a targeted DSB. NHEJ includes two competing pathways known as classic or canonical NHEJ (cNHEJ) and alternative NHEJ (aNHEJ). The cNHEJ pathway requires XRCC4 and DNA ligase IV. The aNHEJ pathway includes MMEJ. The cleaved DSB ends are ligated by cellular DNA repairing machineries using either the precise pathway of cNHEJ or the mutagenic pathway of MMEJ.

Since the advent of Cas9-mediated genome editing, it has long been assumed that Cas9 cleaves the targeting DNA duplex at the −3 position upstream of the PAM site, generating blunted DSB ends with no overhang (Figure 1D; Gasiunas et al., 2012; Jinek et al., 2012). In contrast to the earlier finding that Cas9 has potential exonuclease activity, *in silico* molecular dynamics modeling and *in vitro* high-throughput sequencing suggest that Cas9 cleaves the non-complementary strand at the −4 position upstream of the PAM site (Kim et al., 2016; Palermo et al., 2016; Zuo and Liu, 2016). In addition, *in vitro* cleavage of dsDNA, whose non-complementary strand is labeled at the 3′ ends, reveals both blunted and cohesive Cas9 cleavages (Shou et al., 2018; Stephenson et al., 2018). Specifically, *in vitro* cleavage of dsDNA duplex with the 3′-biotin-labeled non-complementary strand reveals flexible cleavages at the −4 and −3 positions upstream of the PAM site (Shou et al., 2018). Finally, deep sequencing of *in vitro* Cas9-cleaved products reveals flexible cleavages of the non-complementary strand at the −6, −5, −4, and −3 positions upstream of the PAM site but the exact cleavage of the complementary strand at the −3 position (Shi et al., 2019). Collectively, these studies clearly show that Cas9 endonucleolytically cleaves the non-complementary strand at the −6, −5, −4, and −3 positions *in vitro*, generating cohesive DSB ends with 1‒3-nt 5′ overhangs as well as blunted ends (Figure 3A).

### Cohesive Cas9 cleavage in vivo

Overwhelming evidence suggests cohesive Cas9 cleavage *in vivo*. First, the predicted metal coordination distance to the −3 phosphate is much larger than expected for the typical RuvC catalysis (Figure 3B; Chen and Doudna, 2017). Second, Cas9-mediated nucleotide insertions at junctions of DNA fragment editing are strongly biased toward nucleotides at the −6, −5, and −4 positions upstream of the PAM site *in vivo* (Figure 3A;  Shou et al., 2018; Shi et al., 2019). Finally, by engineering the Cas9 hinge regions located between the HNH and RuvC nuclease domains, rationally designed Cas9 variants display R-loop-dependent alterations of the scissile profile of the non-complementary strand *in vivo* (Figure 3A;  Shou et al., 2018). Taken together, these studies suggest that Cas9 cleaves targeting DNA duplex with flexibility on the non-complementary strand, resulting in DSB ends with 5′ overhangs.

### Mechanism of cohesive Cas9 cleavage

Cas9 RuvC and HNH nuclease domains cleave non-complementary and complementary strands via putative two-metal-ion and one-metal-ion mechanisms, respectively (Jinek et al., 2014; Nishimasu et al., 2014; Chen and Doudna, 2017). In both the two-metal-ion and one-metal-ion mechanisms, nucleophilic attack is always in-line from the 5′ site of the phosphodiester bond, resulting in 5′ phosphate and 3′ hydroxyl groups (Figure 3B; Yang, 2010). Whereas one magnesium ion coordinates Cas9 HNH active sites to the scissile phosphate at exactly the −3 position upstream of NGG PAM after a large conformational change, two magnesium ions coordinate Cas9 RuvC active sites to the scissile phosphate at positions further upstream of PAM, resulting in flexible Cas9 cleavages with variable staggered 5′ overhangs.

### After cutting—DSB repair pathways

DNA damage response pathways are activated after Cas9 cleavage to repair the resulting DSBs. The repair of mammalian DSBs involves three possible pathways: HR, canonical non-homologous end-joining (cNHEJ), and alternative non-homologous end-joining (aNHEJ) that includes microhomology-mediated end joining (MMEJ) (Figure 3C; Chang et al., 2017). In mammalian cells, when a template donor is available, the HR repair pathway is used to achieve precise genome editing, including insertion or replacement of specific sequences. However, the low efficiency of HR limits its usage (Ceccaldi et al., 2016a). When no donor is provided, both cNHEJ and aNHEJ (Figure 3C) are predominant pathways for repairing DSBs introduced by Cas9.

In the cNHEJ repair pathway, the Ku70‒Ku80 heterodimer recognizes DSB ends to protect them from being processed by resection nucleases (Figure 3C; Deriano and Roth, 2013). The DNA-dependent protein kinase catalytic subunit (DNA-PKcs) and the endonuclease Artemis are then recruited to the Ku-DNA ends. They form an Artemis‒PK‒Ku complex at the DSB ends. Finally, precise ligations of the two DSB ends are catalyzed by the ligase IV‒XRCC4‒XLF complex (Deriano and Roth, 2013). Thus, cNHEJ is an accurate and precise DSB repair pathway (Shou et al., 2018).

The aNHEJ pathway was originally thought to be a backup repair mechanism for cNHEJ and it usually introduces small indels (Figure 3C). If the cNHEJ repair pathway is not available or is disrupted, the DSB ends will be repaired by the aNHEJ pathway, resulting in error-prone large indels or chromosomal rearrangements. Indeed, in species with no cNHEJ pathway, the genomes are prone to chromosomal rearrangements via aNHEJ (Deng et al., 2018).

In the aNHEJ pathway, extensive resections of DSB ends are catalyzed by several resection nucleases including the MRE11–RAD50–NBS1 (MRN) complex (Nijmegen breakage syndrome protein 1 or nibrin). These resections are facilitated by CtBP-interacting protein (CtIP or RBBP8) and FANCD2 (Ceccaldi et al., 2016b; Chang et al., 2017; Shou et al., 2018). The resection exposes single-stranded DNA (ssDNA) overhangs that could be annealed by complementary base pairing. The annealed DSB ends are then ligated by XRCC1 and DNA ligase III of the aNHEJ pathway, generating indels (Chang et al., 2017). Thus, cNHEJ- and aNHEJ-mediated DNA repairs either join the DSB ends directly or modify them slightly, resulting in precise ligation or small indels, respectively (Figure 3C).

### Random vs. non-random indels

Initial gene editing by CRISPR indicates that prevalent random indels are induced by Cas9 cleavage programmed with single sgRNAs in heterologous systems (Cho et al., 2013; Cong et al., 2013; Jinek et al., 2013; Mali et al., 2013). Similarly, random small indels at the junctions of chromosomal rearrangements—or at the Cas9 cleavage site for the so-called scarring—are also introduced by DNA fragment editing with Cas9 reprogrammed with dual sgRNAs (Canver et al., 2014; Kraft et al., 2015; Li et al., 2015a). These random indels likely result from the NHEJ repair pathway (Figure 3C; Jiang and Marraffini, 2015; Huang and Wu, 2016).

**Figure 4:**
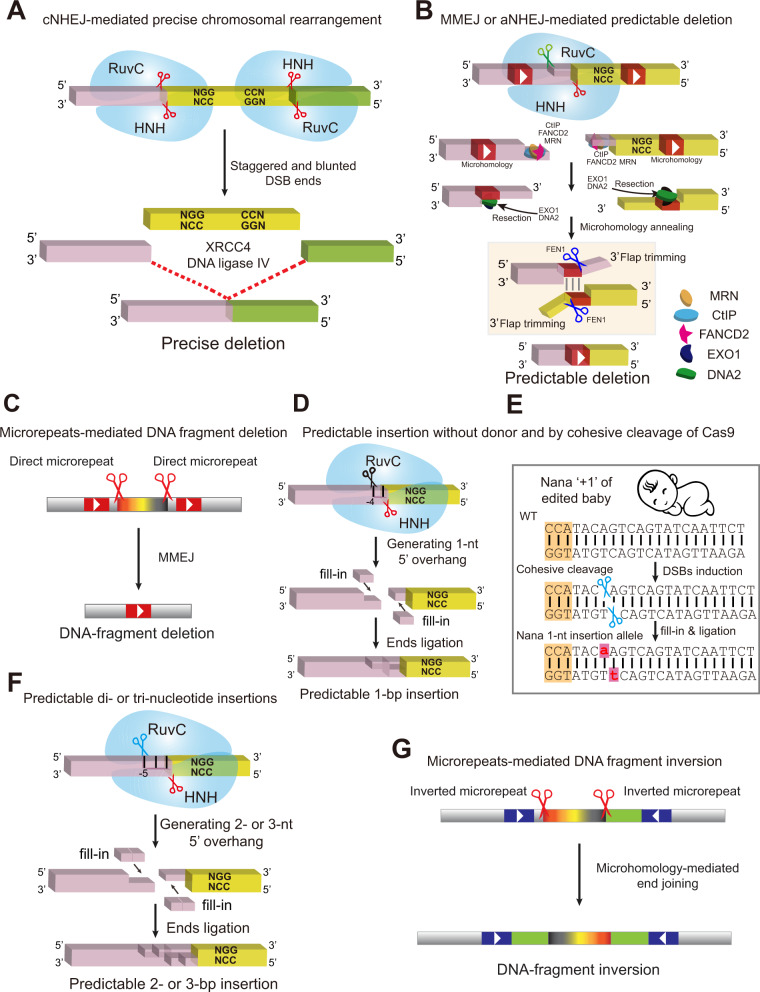
Mechanisms of precise and predictable CRISPR/Cas9 genome editing. (**A**) Precise chromosomal rearrangements by DNA fragment editing. cNHEJ-mediated precise DNA fragment deletion could be generated through direct ligation by XRCC4‒DNA ligase IV of the two staggered or blunted DSB ends from Cas9 cleavage with NGG‒CCN PAM configuration. In particular, perturbations of CtIP or FANCD2, two proteins involved in the aNHEJ pathway, enhance the cNHEJ-mediated precise DNA fragment deletion. (**B**) Predictable deletions. The cohesive and blunted DSB ends could be resected by the MRN complex, resulting in 3′ overhangs. This resection process could be facilitated by CtIP and FANCD2 proteins. Further resection by EXO1 and DNA2 nucleases exposes micro-homologous sequences in the vicinity of the cleavage site. Base-pairing between the microhomologous sequences and removal of the two 3′ overhanging flaps by FEN1 generate predictable deletions. (**C**) Large DNA fragment deletion could also be achieved by MMEJ. When there are direct repeats flanking the two cleavage sites by Cas9 with dual sgRNAs, MMEJ-mediated repair could induce deletion of the intervening sequences between the two direct repeats (rather than between the two cleavage sites through cNHEJ repair pathway). (**D**) Predictable single-nucleotide insertions. Cleavage at the −4 position by Cas9 generates cohesive DSB ends with 1-nt 5′ overhangs. Fill-in and ends ligation by cellular repair machineries result in predictable 1-bp insertions, which are the duplication of the −4 nucleotide. (**E**) The Nana ‘+1’ allele of the human *CCR5* gene in the CRISPR-edited baby probably results from cohesive Cas9 cleavage at the −4 position of the non-complementary strand. (**F**) Predictable di- or tri-nucleotide insertions. Cleavage at the −5 (or −6) position by Cas9 generates cohesive DSB ends with 2-nt (or 3-nt) 5′ overhangs. Fill-in and ends ligation by cellular repair machineries result in predictable 2-bp insertions, which are the duplication of dinucleotide from the −5 and −4 positions. Thus, nucleotide insertions mediated by Cas9 reprogrammed with single sgRNAs manifest as tandem repeats. Finally, nucleotide insertions mediated by Cas9 reprogrammed with dual sgRNAs at various junctions of chromosomal rearrangements are generated by filled-in of cohesive DSB ends. (**G**) Predictable DNA fragment inversion. Large DNA fragment inversion could also be achieved by MMEJ. When there are microhomologous inverted repeats flanking the cleavage sites by Cas9 with dual sgRNAs, MMEJ-mediated repair can induce predictable inversion of the intervening sequence between the inverted repeats (rather than between two cleavage sites through cNHEJ repair pathway).

Subsequent studies by Cas9 reprogrammed with dual sgRNAs show that, in addition to random indels or scarring at individual cleavage sites and rearranged junctions (Cong et al., 2013; Mali et al., 2013; Wang et al., 2013; Xiao et al., 2013; Canver et al., 2014; Guo et al., 2015; He et al., 2015; Kraft et al., 2015; Li et al., 2015a; Schmieder et al., 2018; Shou et al., 2018; Shi et al., 2019), there are predominant ligations at exactly the −3 positions and precise chromosomal rearrangements (Figure 4A; Canver et al., 2014; Guo et al., 2015; Li et al., 2015a; Huang and Wu, 2016; Zhu et al., 2016b). Moreover, profiling of DNA repair outcomes demonstrates that indels induced by Cas9 programmed with single sgRNAs are non-random and are related to sequences of the protospacer (van Overbeek et al., 2016). Finally, recent studies revealed that editing outcomes by the CRISPR/Cas9 system are precise (Figure 4A) and predictable (Figure 4B;  Allen et al., 2018; Chakrabarti et al., 2018; Shen et al., 2018; Shou et al., 2018; Taheri-Ghahfarokhi et al., 2018; Chen et al., 2019; Iyer et al., 2019; Leenay et al., 2019; Long, 2019; Molla and Yang, 2020).

### Predictable deletions

When homologous sequences near the DSB ends generated by Cas9 with single sgRNAs are direct repeats, small deletions could be generated via the MMEJ pathway (Figure 4B; McVey and Lee, 2008; Shou et al., 2018). Specifically, if resections expose short complementary sequences within 3′ overhangs, they will form a DNA duplex and the 3′ flap will be cleaved by flap endonuclease 1 (FEN1), resulting in predictable deletions (Figure 4B; Iyer et al., 2019). Similarly, when direct repeats flank the two cleavage sites of Cas9 targeted by dual sgRNAs, the intervening sequences could be deleted via the MMEJ pathway (Figure 4C; McVey and Lee, 2008; Shou et al., 2018).

## Predictable nucleotide insertions at editing junctions

CRISPR-editing technologies are moving forward at lightning speed. It used to be thought of as uncontrollable or unpredictable but now is considered predictable through machine learning approaches. For example, base-editing outcomes have recently been shown to be predictable (Arbab et al., 2020). In this section, we focus on predictable nucleotide insertions based on the mechanistic understanding of cohesive or staggered Cas9 cleavage. In particular, the cohesive Cas9 cleavage mechanism has a profound impact on gene-editing outcomes of the CRISPR system in a wide variety of scenarios and species. If Cas9 cleavage ends with single-nucleotide 5′ overhangs are filled in and ligated, it will result in duplications of the −4 nucleotide (Table 2). Similarly, if Cas9 cleavage ends with 2-nt overhangs are filled in and ligated, it will lead to repetition of the dinucleotide of the −5 and −4 positions (Table 2). Finally, if Cas9 cleavage ends with 3-nt overhangs are filled in and ligated, it will produce repetition of the trinucleotide of the −6, −5, and −4 positions (Table 2).

**Table 2:** Predictable nucleotide insertions by cohesive Cas9 cleavage with single sgRNAs.

Cell line/organism	Locus	Inserted nt	Reference sequence 5′–3′, mutant sequence 5′–3′	Cohesive cleavage	Reference
Humans	*EMX1*	WT	GAGTCCGAGCAGAAGAAGAA**GGG**	aGAAGGG CTTCCC	Cong et al. (2013)
		(+1)	GAGTCCGAGCAGAAGAA ****a**** GAA ****GGG****		
		
Rats	*Tet1*	WT	ATGAAGACATTGC**TGG**AGACTGTCG	atTGCTGG ACGACC	Li et al. (2013b)
		(+2)	ATGAAGACAT ****at**** TGC **TGG** AGACTGTC		
		
Mice	*Tet2*	WT	GGCTGCTGTCAGGGAGCTCA**TGG**	cTCATGG AGTACC	Wang et al. (2013)
		(+1)	GGCTGCTGTCAGGGAGC **c** TCA **TGG**		
		
K562 cells	*CCR5*	WT	TGACATCAATTATTATACAT**CGG**	aCATCGG GTAGCC	Cho et al. (2013)
		(+1)	TGACATCAATTATTATA **a** CAT **CGG**		
	
	*C4BPB*	WT	AATGACCACTACATCCTCAA**GGG**	tCAAGGG GTTCCC	
		(+1)	AATGACCACTACATCCT **t** CAA **GGG**		
		(+2)	AATGACCACTACATCCT **ct** CAA **GGG**	ctCAAGGG GTTCCC
		
HEK293T cells	*HBB*	WT	CCACGTTCACCTTGCCCCACA**GGG**	cACAGGG TGTCCC	Cradick et al. (2013)
		(+1)	CCACGTTCACCTTGCCCC **c** ACA **GGG**		
		
	*CCR2*	WT	GTGTTCATCTTTGGTTTTGT**GGG**	tTGTGGG ACACCC
		(+1)	GTGTTCATCTTTGGTTT **t** TGT **GGG**	
		(+2)	GTGTTCATCTTTGGTTT **tt** TGT **GGG**	ttTGTGGG ACACCC
		
Yeast	*CAN1*	WT	GATACGTTCTCTATGGAGGA**TGG**	aGGATGG CCTACC	DiCarlo et al. (2013)
		(+1)	GATACGTTCTCTATGGA **a** GGA**TGG**		
		
Zebrafish	*fh*	WT	**CCC**CGGTCGCCATGTACCGCTCC	CCCCGG GGGGCCa	Hwang et al. (2013)
		(+1)	**CCC** CGG **t** TCGCCATGTACCGCTCC		
		
*Arabidopsis*	*AtPDS3*	WT	GGACTTTTGCCAGCCATGGT**CGG**	tGGTCGG CCAGCC	Li et al. (2013a)
		(+1)	GGACTTTTGCCAGCCAT **t** GGT **CGG**		
		
*Nicotiana benthamiana*	*NbPDS3*	WT	GCCGTTAATTTGAGAGTCCA**AGG**	tCCAAGG GGTTCC	Li et al. (2013a)
		(+1)	GCCGTTAATTTGAGAGT **t** CCA **AGG**		
		
Rice	*OsPDS*	WT	GTTGGTCTTTGCTCCTGCAG**AGG**	gCAGAGG GTCTCC	Shan et al. (2013)
		(+1)	GTTGGTCTTTGCTCCTG **g** CAG **AGG**		
		
Rice	*CAO1*	WT	**CCA**AGCTCTTGAGGTGGTCCGGT	CCAAGC GGTTCGa	Miao et al. (2013)
		(+1)	**CCA** AGC **t** TCTTGAGGTGGTCCGGT		
		
Mice intestinal stem cells	*APC locus*	WT	**CCC**TCAAAAGCGTTTTGAGTGCC	CCCTCA GGGAGTt	Schwank et al. (2013)
		(+1)	**CCC** TCA **a** AAAGCGTTTTGAGTGCC		
		
Mice	*EGFP*	WT	GGAGCGCACCATCTTCTTCA**AGG**	tTCAAGG AGTTCC	Shen et al. (2013)
		(+1)	GGAGCGCACCATCTTCT **t** TCA **AGG**		
		
Mice neuron	*GRIN1*	WT	AACCAGGCCAATAAGCGACA**CGG**	gACACGG TGTGCC	Incontro et al. (2014)
		(+1)	AACCAGGCCAATAAGCG **g** ACA **CGG**		
		
K562 cells	*C4BPB*	WT	AATGACCACTACATCCTCAA**GGG**	tCAAGGG GTTCCC	Kim et al. (2014)
		(+1)	AATGACCACTACATCCT **t** CAA **GGG**		
		(+3)	AATGACCACTACATCCT **cct** CAA **GGG**	cctCAAGGG GTTCCC	
		
Mice	*NeuN*	WT	**CCT**TCCGGTTCAGGGACCCCGAC	CCTTCC GGAAGGc	Platt et al. (2014)
		(+1)	**CCT**TCC**g** GGTTCAGGGACCCCGAC		
		(+2)	**CCT** TCC **gg** GGTTCAGGGACCCCGAC	CCTTCC GGAAGGcc	
Murine liver	*Pten*	WT	AGATCGTTAGCAGAAACAAA**AGG**	cAAAAGG TTTTCC	Xue et al. (2014)
		(+1)	AGATCGTTAGCAGAAAC **c** AAA **AGG**		
		
	*P53*	WT	GCCTCGAGCTCCCTCTGAGCC**AGG**	aGCCAGG CGGTCC	
		(+1)	GCCTCGAGCTCCCTCTGA **a** GCC **AGG**	
		
Mice	*Fgf10*	WT	**CCA**CCAACTGCTCTTCTTCCTCC	CCACCA GGTGGTt	Yasue et al. (2014)
		(+1)	**CCA** CCA **a** ACTGCTCTTCTTCCTCC		
		
Mice	*Tyr*	WT	GGGTGGATGACCGTGAGTCC**TGG**	gTCCTGG AGGACC	Fujii et al. (2014)
		(+1)	GGGTGGATGACCGTGAG **g** TCC **TGG**		
		
Mice	*Tet1*	WT	GGCTGCTGTCAGGGAGCTCA**TGG**	cTCATGG AGTACC	Horii et al. (2014)
		(+1)	GGCTGCTGTCAGGGAGC **c** TCA **TGG**		
		
*Drosophila*	*singed (sn)*	WT	GCCAGCACAAGTACATGACCG**CGG**	gaCCGCGG GGCGCC	Lee et al. (2014b)
		(+2)	GCCAGCACAAGTACATGA **ga** CCG **CGG**		
		
*Bombyx mori*	*Bmku70*	WT	GCCATTGGCGCCACCTAACA**TGG**	aACATGG TGTACC	Ma et al. (2014)
		(+1)	GCCATTGGCGCCACCTA **a** ACA **TGG**		
		
Goat fibroblast	*Prp*	WT	AACCGCTATCCACCTCAGGG**AGG**	aGGGAGG CCCTCC	Ni et al. (2014)
		(+1)	AACCGCTATCCACCTCA **a** GGG **AGG**		
		
Monkey	*Ppar-g*	WT	**CCC**TTCACTACTGTTGACTTCTC	CCCTTC GGGAAGt	Niu et al. (2014)
		(+1)	**CCC** TTC **a** ACTACTGTTGACTTCTC		
		
HEK293T cells	*CCR5*	WT	TGACATCAATTATTATACAT**CGG**	aCATCGG GTAGCC	Ramakrishna et al. (2014)
		(+1)	TGACATCAATTATTATA **a** CAT **CGG**		
		
Mice	*Tyr*	WT	**CCT**ATCGGCCATAACAGAGACTC	CCTATC GGATAGc	Yen et al. (2014)
		(+1)	**CCT** ATC **g** GGCCATAACAGAGACTC		
		
Rats	*Tyr*	WT	TTTCCAGGATTATGTAATAG**TGG**	aTAGTGG ATCACC	Yoshimi et al. (2014)
		(+1)	TTTCCAGGATTATGTAA **a** TAG **TGG**		
		(+2)	TTTCCAGGATTATGTAA **aa** TAG **TGG**	aaTAGTGG ATCACC	
		
Mice	*Them2*	WT	**CCT**TAGTGGACAGCATCTCGACC	CCTTAG GGAATCa	Zhu et al. (2014)
		(+1)	**CCT** TAG **t** TGGACAGCATCTCGACC		
		
Mice	*Pitx1*	WT	**CCT**CACTAGAGTACAGGTGTGAA	CCTCAC GGAGTGa	Kraft et al. (2015)
		(+1)	**CCT** CAC **t** TAGAGTACAGGTGTGAA		
		
HCT116 cells	*HPRT gene*	WT	**CCA**GACTGTAAGTGAATTACTTT	CCAGAC GGTCTGa	Liao et al. (2015b)
		(+1)	**CCA** GAC **t** TGTAAGTGAATTACTTT		
		
HCT116 cells	*Trex1*	WT	**CCG**TGTGCGAGTCTGGAGGGGAC	CCGTGT GGCACAc	
		(+1)	**CCG** TGT **g** GCGAGTCTGGAGGGGAC	
		
Zebrafish	*urod*	WT	AGTTCAGGGAATCACGGGCA**GGG**	gGCAGGG CGTCCC	Ablain et al. (2015)
		(+1)	AGTTCAGGGAATCACGG **g** GCA **GGG**		
		
*Nicotiana benthamiana*	*Tomato yellow leaf curl virus*	WT	GGCCATCCGTATAATATTAC**CGG**	tTACCGG ATGGCC	Ali et al. (2015)
		(+1)	GGCCATCCGTATAATAT **t** TAC **CGG**		
		
Murine myeloid progenitor cells	*Bim*	WT	GACAATTGCAGCCTGCTGAG**AGG**	tGAGAGG CTCTCC	Aubrey et al. (2015)
		(+1)	GACAATTGCAGCCTGCT **t** GAG **AGG**		
		(+2)	GACAATTGCAGCCTGCT **ct** GAG **AGG**	ctGAGAGG CTCTCC	
		
Soybean	*GmFEI2*	WT	GTTGGACCTATACCTGCTGA**TGG**	cTGATGG ACTACC	Cai et al. (2015)
		(+1)	GTTGGACCTATACCTGC **c** TGA **TGG**		
Tobacco	*NtPDS*	WT	GAGGCAAGAGATGTCCTAGG**TGG**	tAGGTGG TCCACC	Gao et al. (2015)
		(+1)	GAGGCAAGAGATGTCCT **t** AGG **TGG**		
		
Ghost cells	*CXCR4*	WT	GAAGAAACTGAGAAGCATGA**CGG**	aTGACGG ACTGCC	Hou et al. (2015)
		(+1)	GAAGAAACTGAGAAGCA **a** TGA **CGG**		
		
Jurkat T cells	*CXCR4*	WT	GTTCCAGTTTCAGCACATCA**TGG**	aTCATGG AGTACC	
		(+1)	GTTCCAGTTTCAGCACA **a** TCA **TGG**	
		
Barley (*Hordeum vulgare*)	*HvPM19*	WT	GCTCTCCACTCTGGGCTCTT**CGG**	tCTTCGG GAAGCC	Lawrenson et al. (2015)
		(+1)	GCTCTCCACTCTGGGCT **t** CTT **CGG**		
		
HEK293 cells	*GFP*	WT	GTCGCCACCATGGTGAGCAA**GGG**	gCAAGGG GTTCCC	Liao et al. (2015a)
		(+1)	GTCGCCACCATGGTGAG **g** CAA **GGG**		
		
	*LTR*	WT	GGGAGCTCTCTGGCTAACTA**GGG**	aCTAGGG GATCCC	
		(+1)	GGGAGCTCTCTGGCTAA **a** CTA **GGG**	
		
Human intestinal organoids	*SMAD4*	WT	**CCA**CCAAAACGGCCATCTTCAGC	CCACCA GGTGGTt	Matano et al. (2015)
		(+1)	**CCA** CCA **a** AAACGGCCATCTTCAGC		
		
Soybean	*Glyma06g14180*	WT	GTGAAATTAACCAGCTGCAG**TGG**	gCAGTGG GTCACC	Sun et al. (2015)
		(+1)	GTGAAATTAACCAGCTG **g** CAG **TGG**		
		
Mice	*Pten*	WT	**CCA**TCATCAAAGAGATCGTTAGCA	CCATCA GGTAGTa	Weber et al. (2015)
		(+1)	**CCA** TCA **t** TCAAAGAGATCGTTAGCA		
		
*Nicotiana attenuata*	*AOC*	WT	CAAAAGACTGTCAATTCCCT**TGG**	cCCTTGG GGAACC	Woo et al. (2015)
		(+1)	CAAAAGACTGTCAATTC **c** CCT **TGG**		
		
*Arabidopsis*	*BRI1*	WT	TTGGGTCATAACGATATCTC**TGG**	tCTCTGG GAGACC	Yan et al. (2015)
		(+1)	TTGGGTCATAACGATAT **t** CTC **TGG**		
		
*Nicotiana benthamiana*	*NbIspH*	WT	GAATGGATATGAGTACACTT**GGG**	aCTTGGG GAACCC	Yin et al. (2015)
		(+1)	GAATGGATATGAGTACA **a** CTT **GGG**		
		
Mice	*Kcnj13*	WT	**CCT**GCGATGGACAGCAGTAATTG	CCTGCG GGACGCt	Zhong et al. (2015)
		(+1)	**CCT** GCG **a** ATGGACAGCAGTAATTG		
		
Mice	*Nf1*	WT	AGTCAGCACCGAGCACAACA**AGG**	aACAAGG TGTTCC	Zuckermann et al. (2015)
		(+1)	AGTCAGCACCGAGCACA **a** ACA **AGG**		
		
	*Pten*	WT	AAAGACTTGAAGGTGTATAC**AGG**	aTACAGG ATGTCC	
		(+1)	AAAGACTTGAAGGTGTA **a** TAC **AGG**		
		
	*Trp53*	WT	ACAGCCATCACCTCACTGCA**TGG**	tGCATGG CGTACC
		(+1)	ACAGCCATCACCTCACT **t** GCA **TGG**	
		
HEK293T, K562, HCT116	*Non*-*coding region*	WT	GGCAGTGCAGATGAAAAACT**GGG**	aACTGGG TGACCC	van Overbeek et al. (2016)
		(+1)	GGCAGTGCAGATGAAAA **a** ACT **GGG**		
		
HEK293T, K562	*Chr1:65349091*	WT	GAGGAGCTCCAAGAAGACTG**AGG**	aCTGAGG GACTCC	
		(+1)	GAGGAGCTCCAAGAAGA **a** CTG **AGG**	
		
*Yarrowia lipolytica*	*PEX10*	WT	GCCCAGCCCGGAAACATGGA**AGG**	tGGAAGG CCTTCC	Gao et al. (2016)
		(+1)	GCCCAGCCCGGAAACAT **t** GGA **AGG**		
		(+2)	GCCCAGCCCGGAAACAT **at** GGA **AGG**	atGGAAGG CCTTCC	
		
Murine HSPCs	*Eed*	WT	TGCTTGCATTGGGCAATC**AGG**	aATCAGG TAGTCC	Gundry et al. (2016)
		(+1)	TGCTTGCATTGGGCA **a** ATC **AGG**		
*Taraxacum*	*Fructan 1-fructosyltransferase*	WT	ACAACCCGTACGCACCAATT**TGG**	aATTTGG TAAACC	Iaffaldano et al. (2016)
		(+1)	ACAACCCGTACGCACCA **a** ATT **TGG**		
		
Apple	*PDS*	WT	ATGGCTTGAGCGTAAAAGAC**TGG**	aGACTGG CTGACC	Nishitani et al. (2016)
		(+1)	ATGGCTTGAGCGTAAAA **a** GAC **TGG**		
		
*Phaeodactylum tricornutum* cells	*CpSRP54*	WT	CCGCCCTTCGTGAAGTACGT**CGG**	aCGTCGG GCAGCC	Nymark et al. (2016)
		(+1)	CCGCCCTTCGTGAAGTA **a** CGT **CGG**		
		
Chardonnay	*IdnDH*	WT	GGGGAAAGGAGGCAACTCTG**AGG**	tCTGAGG GACTCC	Ren et al. (2016)
		(+1)	GGGGAAAGGAGGCAACT **t** CTG **AGG**		
		
Maize immature embryo cells	*liguleless1 (LIG)*	WT	ATACGCGTACGCGTACGTGTG**AGG**	tGTGAGG CACTCC	Svitashev et al. (2016)
		(+1)	ATACGCGTACGCGTACGT **t** GTG **AGG**		
		
SNU719 cells	*EBV genomic locus of BART5*	WT	**CCT**CAAGGTGAATATAGCTGCCC	CCTCAA GGAGTTc	van Diemen et al. (2016)
		(+1)	**CCT** CAA **g** GGTGAATATAGCTGCCC		
		
HEK293 cells	*GFP*	WT	GGGCGAGGAGCTGTTCACCG**GGG**	aCCGGGG GGCCCC	Yin et al. (2016)
		(+1)	GGGCGAGGAGCTGTTCA **a** CCG **GGG**		
		
Wheat	*TaGW2*	WT	**CCT**CTAGAAATGCCCCATCCTG	CCTCTA GGAGATc	Zhang et al. (2016)
		(+1)	**CCT** CTA **g** GAAATGCCCCATCCTG		
		
Maize	*PSY1*	WT	GAGACTTGAGGATCTGTTCA**CGG**	tTCACGG AGTGCC	Zhu et al. (2016a)
		(+1)	GAGACTTGAGGATCTGT **t** TCA **CGG**		
		
Gal4EED HEK293	*firefly luciferase*	WT	AAGAGATACGCCCTGGTTCC**TGG**	gtTCCTGG AGGACC	Daer et al. (2017)
		(+2)	AAGAGATACGCCCTGGT **gt** TCC **TGG**		
		
Chicken DF-1 fibroblasts	*Pax7*	WT	**CCA**TGGCTGATGACCAAGATCTG	CCATGG GGTACCg	Gandhi et al. (2017)
		(+1)	**CCA** TGG **c** CTGATGACCAAGATCTG		
		
Cotton	*GhPDS*	WT	GAAGCGAGAGATGTTCTAGG**TGG**	tAGGTGG TCCACC	Gao et al. (2017)
		(+1)	GAAGCGAGAGATGTTCT **t** AGG **TGG**		
		
Mice liver	*Ldlr*	WT	TGCTGCTGGCCAAGGACATG**CGG**	cATGCGG TACGCC	Jarrett et al. (2017)
		(+1)	TGCTGCTGGCCAAGGAC **c** ATG **CGG**		
		
Bread wheat	*TaGW2*	WT	**CCT**CTAGAAATACCCCATCCTG	CCTCTA GGAGATc	Liang et al. (2017)
		(+1)	**CCT** CTA **g** GAAATACCCCATCCTG		
		
TZM-bl cells	*CXCR4*	WT	GCTTCTACCCCAATGACTTG**TGG**	cTTGTGG AACACC	Liu et al. (2017b)
		(+1)	GCTTCTACCCCAATGAC **c** TTG **TGG**		
		
Mice	*Kcnk13*	WT	**CCT**GAACGAGGACAACGCGCGCT	CCTGAA GGACTTg	Mianne et al. (2017)
		(+1)	**CCT** GAA **c** CGAGGACAACGCGCGCT		
		
Hexaploid Camelina sativa	*FAD2*	WT	TCAAGGCTGTGTCCTAAC**CGG**	tAACCGG TTGGCC	Morineau et al. (2017)
		(+1)	TCAAGGCTGTGTCCT **t** AAC **CGG**		
		
T cells	*TCR a*	WT	TGTGCTAGACATGAGGTCTA**TGG**	tCTATGG GATACC	Ren et al. (2017)
		(+1)	TGTGCTAGACATGAGGT **t** CTA **TGG**		
		
Watermelon	*ClPDS*	WT	ATGCCGCTAGAGTGGTGCC**CGG**	tGCCCGG CGGGCC	Tian et al. (2017)
		(+1)	ATGCCGCTAGAGTGGT **t** GCC **CGG**		
		
MCF-7 cells	*HER2*	WT	GGGCATGGAGCACTTGCGAG**AGG**	cGAGAGG CTCTCC	Wang and Sun (2017)
		(+1)	GGGCATGGAGCACTTGC **c** GAG **AGG**		
		
Reef-building coral	*RFP*	WT	GTCTTCACTGAATATCCTCA**AGG**	cTCAAGG AGTTCC	Cleves et al. (2018)
		(+1)	GTCTTCACTGAATATCC **c** TCA **AGG**		
Solanaceae crop *Physalis pruinosa*	*Ppr-SP*	WT	**CCT**TCCTTAGTCACCTCTAAACC	CCTTCC GGAAGGa	Lemmon et al. (2018)
		(+1)	**CCT** TCC **t** TTAGTCACCTCTAAACC		
		
K562 cells	*ND*	WT	GCATCGGCCTGAAAGCAGTG**AGG**	aGTGAGG CACTCC	Allen et al. (2018)
		(+1)	GCATCGGCCTGAAAGCA **a** GTG **AGG**		
		
HPS1 B-LCL cells	*HPS1*	WT	CAGCAGGGGAGGCCCCCAGC**AGG**	cAGCAGG TCGTCC	Iyer et al. (2019)
		(+1)	CAGCAGGGGAGGCCCCC **c** AGC **AGG**		

### Predictable single-nucleotide insertions at single cutting sites

Extensive studies have shown that Cas9-mediated single-nucleotide insertions at repair junctions in budding yeast, mouse ESCs, mammalian cell lines, and mice are predictable (Figure 4D;  Chakrabarti et al., 2018; Kalhor et al., 2018; Lemos et al., 2018; Shen et al., 2018; Shou et al., 2018; Taheri-Ghahfarokhi et al., 2018; Chen et al., 2019; Gisler et al., 2019; Leenay et al., 2019). When Cas9 reprogrammed with single sgRNAs cleaves the non-complementary strand at the −4 position, it will generate two cohesive ends with 1-nt 5′ overhangs, which could be filled-in by an unknown polymerase (Figure 4D). The two filled-in DSB ends are then ligated directly, generating single-nucleotide insertion which is the duplication of the −4 nucleotide upstream of PAM (Figure 4D).

This ligation mechanism is via the cNHEJ pathway since blocking XRCC4 results in a significant decrease of precise ligation in DNA fragment editing (Shou et al., 2018). In addition, knocking down of DNA ligase IV leads to a significant decrease of precise DNA-fragment-deletion efficiency, suggesting that cNHEJ is an error-free DNA repair pathway (Shou et al., 2018). Therefore, numerous cases of 1-bp insertions, which were reported as random insertions, actually result from Cas9 cohesive cleavage at the −4 position (Table 2). For example, the Nana ‘+1’ allele of *CCR5* of the unethically edited baby (Ryder, 2018) is probably generated by cohesive Cas9 cleavage at the −4 position, resulting in two DSB ends with 1-nt 5′ overhang, which are then filled in and ligated precisely (Figure 4E). All in all, gene editing via Cas9 cohesive cleavage at the −4 position generates predictable 1-bp insertions (Table 2).

### Dinucleotide and trinucleotide insertions at single cutting sites

If Cas9 RuvC domain cleaves the non-complementary strand at the −5 or −6 position upstream of PAM, it will generate two cohesive DSB ends each with a dinucleotide or trinucleotide 5′ overhang. After both of them get filled-in, these filled-in ends could be blunt-end ligated via the cNHEJ pathway. This will generate a dinucleotide or trinucleotide insertion, which is the tandem duplication of the dinucleotide or trinucleotide further upstream of the −3 position of PAM (Table 2; Figure 4F).

### Prominent predictable nucleotide insertions at rearranged junctions of double cutting

Systematic analyses of the inserted nucleotides reveal predictable nucleotide insertions at the junctions of chromosomal rearrangements by Cas9 with dual sgRNAs (Table 3; Shou et al., 2018). Interestingly, the frequency of nucleotide insertions (1, 2, or 3 nt) is much higher at junctions of chromosomal rearrangements by double cutting than that by single cutting (Shi et al., 2019). The reason for the increased insertion frequency at rearranged junctions is that the ligated junctions of chromosomal rearrangement after Cas9 double cleavages cannot be recut. For single Cas9 cleavages, the two cohesive DSB ends are always complementary to each other (Figure 3A). After annealing of the cohesive ends and ligation by cellular repair machineries, it will be recut by Cas9 programmed with the same sgRNA. By contrast, any two DSB ends from chromosomal rearrangements, which have distinct 5′ overhangs, are rarely complementary to each other, and thus cannot be annealed and recut by Cas9 programmed with either of the two original sgRNAs.

**Table 3:** Predictable nucleotide insertions by cohesive Cas9 cleavage with dual sgRNAs.

Cell line/organism	Locus	Editing event	Inserted nt	Reference sequence 5′–3′, mutant sequence 5′–3′	Cohesive cleavage	Reference
Mice	*Hprt*	Large DNA fragment deletion	WT	**CCC**GTCATGCCGACCCGCAGTCC --//-- GAAAAAGTGTTTATTCCTCA**TGG**		Fujii et al. (2013)
			(+1)	**CCC** GTC ------------------//--------------------- **c** TCA**TGG**	cTCATGG AGTACC	
Drosophila	*yellow gene*	Large DNA fragment deletion	WT	**CCT**GATTACCCGAACACTGAACC --//-- GGTTAACATAATCCTACACA**CGG**		Gratz et al. (2013)
			(+4)	**CCT**GAT **tacc** ---------------//------------------------ **CGG**	CCTGAT GGACTAatgg	
Murine erythroleukemia cells	*ND*	Large DNA fragment deletion	WT	TGAGCAGGCACTAGACGGAT**GGG** --/2 kb /-- CACAGAAAGTCTTGATCTCG**GGG**		Canver et al. (2014)
			(+1)	TGAGCAGGCACTAGACG -------------------------------- **c** TCG **GGG**	cTCGGGG AGCCCC	
			(+2)	TGAGCAGGCACTAGACG ------------------------------- **tc** TCG **GGG**	tcTCGGGG AGCCCC	
Liver cancer cell line Huh7.5OC	*CSPG4 gene*	Large DNA fragment deletion	WT	GTCTAGTGAGACGGAGGCG**TGG** --/3.48 kb /-- TGCTGGGAGGAGGTTTGAGA**GGG**		Zhu et al. (2016b)
			(+1)	GTCTAGTGAGACGGAG ----------------------------------- **g** AGA **GGG**	gAGAGGG TCTCCC	
K562 cells	*AAVS1 locus*	Large DNA fragment deletion	WT	**CCC**AGAGACAGTGACCAACCATC --/1.05 kb /-- CTCCCTCCCAGGATCCTCTC**TGG**		Cho et al. (2014)
			(+2)	**CCC**AGA --------------------------------------------- **ct** CTC **TGG**	ctCTCTGG GAGACC	
HEK293T cells	*APC*	Large DNA fragment deletion	WT	**CCA**GCCTGAGTGCTCTGAGCCTC --/2.43 kb /-- GGCCGAAACTCAATTTCCCC**TGG**		Sakuma et al. (2014)
			(+1)	**CCA**GCC ---------------------------------------------- **c** CCC **TGG**	cCCCTGG GGGACC	
Tobacco	*PDS*	Large DNA fragment inversion	Reference	TATAGATGACTGGAAAAAATCA**CCT**GCACC…GCTGCATGGAAAGATGATGA**TGG**	CCTGCA GGACGTg	Gao et al. (2015)
			(+1/+1)	TATAGATGACTGGAAAAAATCA **CCT** GCA **ca** TGA **TGG**	aTGATGG ACTACC	
Rice	*OsYSA*	Large DNA fragment deletion	WT	**CCG**CTTCGGCCGAGGTGGCGCGC --//-- **CCT**CATGAAGGTGCTCGTCGCG		Lowder et al. (2015)
			(+1)	**CCG** CTT **c** ----------------------------GAAGGTGCTCGTCGCG	CCGCTT GGCGAAg	
			(+2)	**CCG** CTT **cg** ---------------------------GAAGGTGCTCGTCGCG	CCGCTT GGCGAAgc	
*Arabidopsis thaliana* protoplasts	*BRI1*	Large DNA fragment deletion	WT	TTTGAAAGATGGAAGCGCGG**TGG** --/201 bp /-- TGAAACTAAACTGGTCCACA**CGG**		Woo et al. (2015)
			(+1)	TTTGAAAGATGGAAGCG ---------------------------------- **c** ACA **CGG**	cACACGG TGTGCC	
Rice	*MPK5*	Large DNA fragment deletion	WT	**CCC**TCCTTGAGGCGACCGGGTTC --/473 bp /-- GAATGCGCAGACTCGTCAGG**AGG**	CCCTCC GGGAGGa	Xie et al. (2015)
			(+1/+1)	**CCC** TCC **t** -------------------------------------------- **c** AGG **AGG**	cAGGAGG TCCTCC	
Human T cells	*hPD-1 locus*	Deletion	WT	**CCG**CTTCCGTGTCACACAACTGCCCAACGGGCGTGACTTCCACATGAGCG**TGG**		Su et al. (2016)
			(+1)	**CCG**CTT ------------------------------------------ **a** GCG **TGG**	aGCGTGG CGCACC	
Cotton	*GhCLA1*	Large DNA fragment deletion	WT	**CCA** AGCAAATCGGTGGGCCTATG-- /415 bp / --GTGAAGTTCGATCCGGCAAG **TGG**		Wang et al. (2018)
			(+1)	**CCA** AGC **a** --------------------------------------------- AAG**TGG**	CCAAGC GGTTCGt	
HEC-1-B cells	*Pcdh*	Large DNA fragment deletion	WT	GCCACACATCCAAGGCTGAC**AGG** --/1233 bp /-- AGATTTGGGGCGTCAGGAAG**TGG**		Shou et al. (2018)
			(+1)	GCCACACATCCAAGGCT ----------------------------------- gAAG **TGG**	gAAGTGG TTCACC	
			(+2)	GCCACACATCCAAGGCT ---------------------------------- ggAAG **TGG**	ggAAGTGG TTCACC	
			(+3)	GCCACACATCCAAGGCT --------------------------------- aggAAG **TGG**	aggAAGTGG TTCACC	
	*β-globin*	Large DNA fragment deletion	WT	ACCCAATGACCTCAGGCTGT**AGG** --/6277 bp /-- TCACTTGTTAGCGGCATCTG**TGG**		
			(+1)	ACCCAATGACCTCAGGC ---------------------------- --------- tCTG **TGG**	tCTGTGG GACACC	
			(+2)	ACCCAATGACCTCAGGC ---------------------------------- atCTG **TGG**	atCTGTGG GACACC	
			(+3)	ACCCAATGACCTCAGGC --------------------------------- catCTG **TGG**	catCTGTGG GACACC	

**Figure 5:**
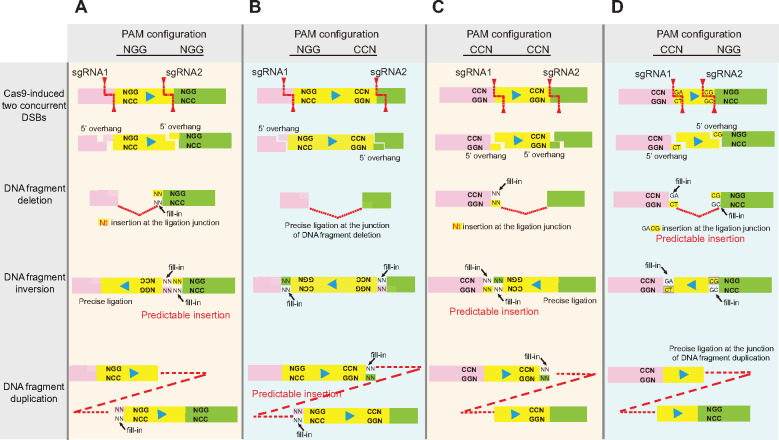
Precise and predictable Cas9-mediated nucleotide insertions at the junctions of chromosome arrangements for the four PAM configurations by Cas9 with dual sgRNAs. (**A**) In the NGG‒NGG PAM configuration, the nucleotide insertions at the downstream junctions of DNA fragment inversion could be predicted based on combined flexible cleavage profiles of Cas9 with sgRNA1 and sgRNA2. However, the upstream junctions of DNA fragment inversion in the NGG‒NGG PAM configuration are precise. (**B**‒**D**) Similarly, the nucleotide insertions at the junctions of DNA fragment duplication (**B**), at the upstream junctions of DNA fragment inversion (**C**), and at the junctions of DNA fragment deletion (**D**) are predictable in the NGG‒CCN (**B**), CCN‒CCN (**C**), and CCN‒NGG (**D**) PAM configurations, respectively. In addition, the ligations at the junctions of DNA fragment deletion (**B**), at the downstream junctions of DNA fragment inversion (**C**), and at the junctions of DNA fragment duplication (**D**) are precise in the NGG‒CCN (**B**), CCN‒CCN (**C**), and CCN‒NGG (**D**) PAM configurations, respectively.

There are barely any 2- or 3-bp insertions with Cas9 reprogrammed with single sgRNAs (Figure 4F; Allen et al., 2018; Shen et al., 2018; Chen et al., 2019; Leenay et al., 2019). In addition, Cas9 reprogrammed with single sgRNA shows significantly higher frequency of 1-bp insertions than 2- or 3-bp insertions (Chen et al., 2019; Shi et al., 2019). The reason that 2- or 3-bp insertions with Cas9 guided by single sgRNAs are much less observable (Allen et al., 2018; Shen et al., 2018; Leenay et al., 2019; Shi et al., 2019) than by dual sgRNAs (Shou et al., 2018; Shi et al., 2019; Figure 4F) is that the annealing efficiencies of 2- or 3-bp overhangs after Cas9 single cleavages are much higher than that of 1-bp overhangs, and thus the repaired 2- or 3-bp cohesive overhangs are more frequent to be recut. Overall, predictable nucleotide insertions are easily observed at junctions of chromosome rearrangements by Cas9 with dual sgRNAs (Figure 5; Shou et al., 2018; Shi et al., 2019).

## Toward precise and predictable genome editing

In order to achieve precise and predictable genome editing, the Cas9 endonuclease effector needs first to be located precisely to a targeting site. Once targeted to a genome site, the Cas9 effector can make a predictable modification on the sequences of the targeting site. Novel derivative gene-editing systems such as base editing and prime editing are developed rapidly (Anzalone et al., 2020; Yang and Chen, 2020). The base-editing system is achieved by fusing dCas9 with a nucleobase deaminase such as cytidine deaminases of the APOBEC/AID family or adenosine deaminase (Komor et al., 2016; Gaudelli et al., 2017). The prime-editing system is achieved by fusing H840A Cas9 with a reverse transcriptase and also fusing sgRNA with designed sequences functioning as a priming RNA template for reverse transcription, so-called prime-editing guide RNA or pegRNA (Anzalone et al., 2019). Both of these new gene-editing systems have advantages of precise editing without the requirement of DNA donor templates and DSBs. In this section, we focus only on precise and predictable genome editing derived from the mechanistic understanding of the Cas9 catalysis.

### Factors influencing CRISPR genome editing

Various factors influence the complexity of DNA repair outcomes, including the type of DNA repair pathways chosen by host cells, the diversity of DSB ends from Cas9 cleavage, and the 3D genome sequence context surrounding the DSBs. In particular, inhibiting the aNHEJ pathway by knocking down its component proteins of CtIP or FANCD2 enhances precise DNA fragment deletion since cNHEJ and aNHEJ compete with each other for repair substrates (Figure 3C;  Shou et al., 2018). Conversely, overexpression of CtIP protein facilitates usage of the MMEJ pathway and results in predictable deletions (Figure 4B; Nakade et al., 2018). In addition, interplays between structures of DSB ends and cellular repair protein machineries (resection nucleases, polymerases, and ligases) likely determine end-joining patterns. Indeed, DSB polarity influences repair outcomes at the editing junctions of Cas9-induced artificial class switching and translocations in human B cells (So and Martin, 2019).

### Mechanism for computer programs of machine learning

Precise and predictable Cas9-mediated genome editing could be achievable through machine learning. For example, computer programs with machine learning algorithms have been recently developed to predict repair outcomes and to achieve predictable genome editing (Allen et al., 2018; Shen et al., 2018; Chen et al., 2019; Leenay et al., 2019). Specifically, with editing using *Sp*Cas9 with the PAM site of NGG, the presence of a nucleotide of ‘T’ or ‘A’ at the −4 position tends to result in more predictable 1-bp insertions. In contrast, the presence of a nucleotide of ‘G’ at the −4 position tends to generate more predictable deletions. The reason for this deletion preference is related to microhomology between the ‘G’ at the −4 position and the N‘GG’ of the PAM site (Shi et al., 2019).

### Predictable MMEJ-mediated DNA fragment inversion

Short inverted repeats flanking the two cleavage sites induce microhomology-mediated inversion of the intervening sequences. Namely, when homology sequences near the DSB ends are inverted repeats, the intervening sequences can be inverted via the MMEJ pathway (Figure 4G; McVey and Lee, 2008; Li et al., 2015a). Therefore, MMEJ-mediated precise DNA fragment editing may be predicted from microhomologous sequences around the two cleavage sites.

### Toward predictable chromosomal rearrangements

Cas9 programmed with dual sgRNAs induces predictable junctional insertions of DNA fragment editing since specific PAM configurations can generate distinct combinations of DSB ends from cohesive Cas9 cleavages (Figure 5; Shou et al., 2018). For example, in the NGG‒NGG PAM configuration, the flexible cleavage profile of Cas9 with sgRNA2 can be obtained by sequencing rearranged junctions of DNA fragment deletion. Similarly, the flexible cleavage profile of Cas9 with sgRNA1 can be obtained by sequencing rearranged junctions of DNA fragment duplication. The nucleotide insertions at the downstream junctions of DNA fragment inversion can be easily predicted by the combined cleavage profiles of both sgRNAs (Figure 5A). Note that the upstream junctions of DNA fragment inversion for the NGG‒NGG PAM configuration are always precise (Figure 5A). Similarly, the rearranged junctions of DNA fragment deletion (Figure 5B), the downstream junctions of DNA fragment inversion (Figure 5C), and the rearranged junctions of DNA fragment duplication (Figure 5D) are always precise for the NGG‒CCN, CCN‒CCN, and CCN‒NGG PAM configurations, respectively. In addition, the nucleotide insertions at rearranged junctions of DNA fragment duplication, the upstream junctions of DNA fragment inversion, and the rearranged junctions of DNA fragment deletion are predictable for the NGG‒CCN, CCN‒CCN, and CCN‒NGG PAM configurations, respectively (Figure 5B‒D). Understanding the mechanisms of chromosomal rearrangements will facilitate precise and predictable CRISPR DNA fragment editing.

### Chromosomal rearrangement mechanisms in the context of 3D genome

After Cas9 cleavage, the histone H2AX within nucleosomes located in the regions flanking the DSB ends is phosphorylated by the ATM kinase, generating γH2AX (Iacovoni et al., 2010; Lee et al., 2014a). Interestingly, a recent study showed that Cas9 is a genome mutator and induces γH2AX accumulation (Xu et al., 2020). In addition, long-distance chromatin interactions are increased within the γH2AX chromatin domains (Aymard et al., 2017). However, whether these increased chromatin interactions influence the form of the so-called ‘DNA repair foci’ needs further exploration (Marnef and Legube, 2017).

Several recent studies have shown that CTCF participates in DSB repair through its interaction with the repair proteins of BRCA2, RAD51, Mre11, and CtIP (Han et al., 2017; Hilmi et al., 2017; Lang et al., 2017; Hwang et al., 2019). In addition, cohesin inhibits distal DSB end joining (Gelot et al., 2016). Because CTCF and cohesin are known prominent 3D genome architecture proteins (Merkenschlager and Nora, 2016), the recruitment of CTCF and its associated cohesin complex to the regions around DSB ends suggests that 3D genome architecture is closely related to DNA DSB repair.

### 3D motility of DSB ends in the nuclear space

In order to repair and ligate Cas9-induced DSB ends, they need to be brought into close spatial contact in the 3D nuclear space. Nuclear actin may play an important role in DSB motility required for both HR and NHEJ repairs (Caridi et al., 2018). Clustering of DSB ends and formation of a macro-repair center may be a prerequisite for proper chromosomal rearrangements by DNA fragment editing (Jasin and Rothstein, 2013; Aymard et al., 2017).

## Toward precise and predictable 3D genome editing: from 1D to 3D

The higher order chromatin structure is highly dynamic and is regulated by epigenetic processes of DNA methylation, histone modification, and chromatin remodeling, ensuring proper cellular processes such as DNA replication, RNA transcription, and DNA damage repair in response to developmental or physiological signals (Dekker and Mirny, 2016; Hansen et al., 2018; Bickmore, 2019). Structural variations or chromosomal rearrangements affect 3D genome organization and gene expression. Editing of higher order chromatin structures or engineering chromosomal rearrangements to model genome structural variations not only sheds light on the fundamental mechanisms of 3D genome folding but also contributes to our understanding of aberrant 3D genome folding in human diseases (Wang et al., 2019b). Specifically, 3D genome engineering may pave the way to understanding vast GWAS data and CRISPR correction of aberrant alleles may lead to human disease therapy in the future (Qian et al., 2019).

Proximity ligation-based chromosome conformation capture (3C) technologies, in conjunction with high-throughput next-generation sequencing, have led to tremendous progress in understanding 3D genome architecture (Dekker et al., 2002; Rao et al., 2014; Liu et al., 2017a; Tan et al., 2019; reviewed in Denker and de Laat, 2016; Zheng and Xie, 2019). In addition, fluorescence-labeled single-molecule imaging with super-resolution microscopy has shed significant light on the mechanisms of genome folding (Hansen et al., 2018; Sigal et al., 2018). Although genetic methods have long been used to investigate the position-effects variegations of chromatin organization (Lewis, 1950; McClintock, 1950), they have not been widely used to probe 3D genome organization compared to various chromosome conformation capture (3C, 4C, 5C, 6C, 7C, Hi-C, capture-C, etc.) ‘C’ technologies and imaging methods.

### General principles of 3D genome organization

The 3D genomes in the nuclear space are thought to be assembled in a hierarchical manner composed of successive chromosomal territories, compartments or clustering regions, TADs or topological domains, and chromatin loops (Dekker and Mirny, 2016; Dixon et al., 2016; Bickmore, 2019). Briefly, each interphase chromosome occupies a distinct territory. Within a chromosome territory, chromatin fibers are segregated into active and inactive compartments with distinct histone modifications. Chromatin compartments are further divided into TADs or topological domains which are thought to be enriched in long-distance chromatin contacts or loops (Bonev and Cavalli, 2016). Emerging evidence suggests, however, that chromosome compartments are smaller than previously thought and could be the consequences of gene activity (Rowley and Corces, 2018). Nevertheless, chromatin loops are fundamental units of the higher order chromatin structures.

### CRISPR DNA fragment inversion reveals that the locations and relative orientations of CTCF sites determine the directionality of chromatin looping

**Figure 6:**
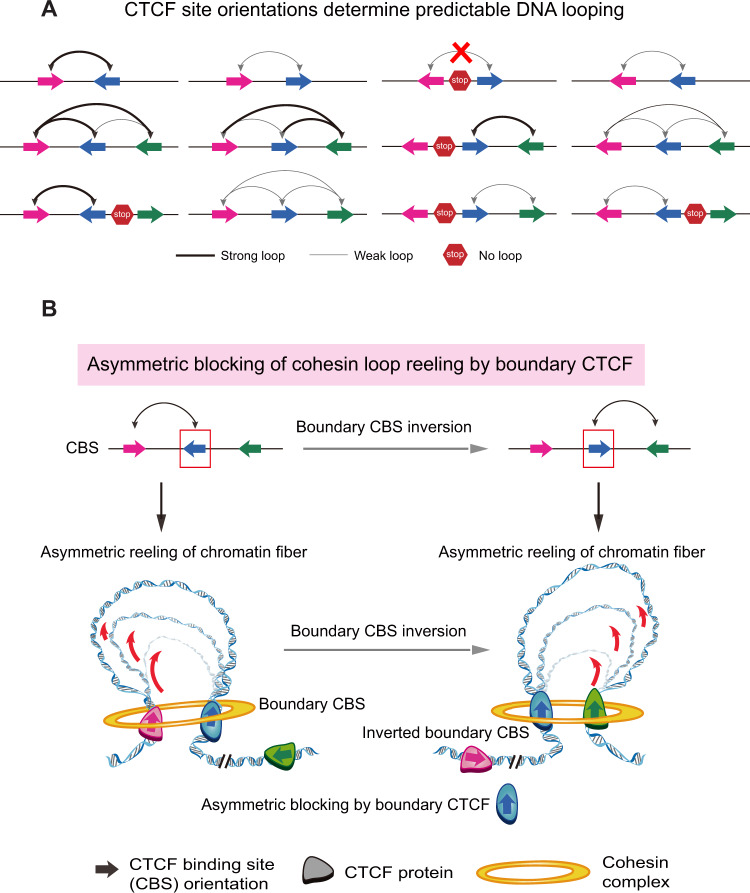
Predictable 3D genome engineering. (**A**) CTCF coding from 1D genomic sequences to 3D genome organization. The topology and strength of chromatin loops can be predicted based on the locations and relative orientations of CTCF sites. (**B**) Schematic of asymmetric ‘loop extrusion’ model revealed by CRISPR inversion of boundary CTCF sites. Genetic manipulation of CTCF sites demonstrates asymmetric blocking of cohesin loop extrusion by directional CTCF binding to oriented CBS elements. Chromatin fibers are compacted by active cohesin ‘loop extrusion’ with ‘two heads’. Cohesin complex reels in chromatin fibers until anchored by oriented CTCF sites. If ‘one head’ of cohesin is anchored by CTCF sites, cohesin can continue to reel in chromatin fibers through the ‘other head’, resulting in so-called asymmetric ‘loop extrusion’.

Inversion of CTCF sites in the protocadherin alpha (*Pcdhα*) and *β-globin* clusters switches the directionality of chromatin looping (Guo et al., 2015; Shou et al., 2018; Jia et al., 2020). Specifically, the causality between orientation of mammalian insulators known as CTCF sites and directionality of long-distance chromatin looping is demonstrated by inverting CTCF sites using CRISPR DNA fragment-editing methods (Figure 6A; Guo et al., 2015; Shou et al., 2018; Lu et al., 2019; Jia et al., 2020). In addition, haplotype variants that alter chromatin looping topology are linked to human disease risks (Tang et al., 2015). In the *Sox2* and *Fbn2* loci, however, reinserting an inverted CTCF site in the original location does not form new chromatin loops (de Wit et al., 2015). Nevertheless, alterations of native chromatin loops have functional consequence on gene expression (de Wit et al., 2015; Guo et al., 2015). Moreover, genome-wide distributions of forward and reverse CTCF sites tend to be located in close 3D spaces (Rao et al., 2014; Guo et al., 2015). Thus, the relative orientations of CTCF sites determine the directionality of chromatin looping across mammalian genomes (Figure 6A). Specifically, there are strong long-distance chromatin interactions between forward and reverse convergent CTCF sites. However, there are weak long-distance chromatin interactions between two tandem CTCF sites in the same orientation. Finally, the configuration of reverse and forward CTCF sites constrains long-distance chromatin interactions between remote elements (Figure 6A). In summary, 3D genome structures could be predicted from 1D nucleotide sequences based on this CTCF-coding mechanism.

### Mechanism of 3D genome folding by cohesin ‘loop extrusion’

The CTCF coding for the 3D genome could be explained by CTCF blocking of cohesin ‘loop extrusion’ along chromatin fibers (Guo et al., 2015; Nichols and Corces, 2015; Sanborn et al., 2015; Fudenberg et al., 2016; Merkenschlager and Nora, 2016; Li et al., 2020b). The current model for the formation of TADs or topological domains is the cohesin sliding-mediated ‘loop extrusion’ (Banigan and Mirny, 2020). Specifically, CTCF helps to establish TADs boundaries by stalling the sliding of cohesin on DNA fibers and thus facilitates chromatin loop formations by ‘two-headed’ cohesin complex (Jia et al., 2020). Therefore, the cohesin complex can bring distant DNA elements into close spatial contact by the so-called active ‘loop extrusion’, which requires ATP as an energy source (Davidson et al., 2019; Kim et al., 2019). The genome-wide colocalization of CTCF and cohesin as well as a strong tendency of long-distance chromatin interactions between forward–reverse convergent CTCF sites provide strong evidence for CTCF stalling of cohesin ‘loop extrusion’ (Parelho et al., 2008; Wendt et al., 2008; Rao et al., 2014; Guo et al., 2015). In addition, consistent with the model of cohesin ‘loop extrusion’, deletion of WAPL, a cohesin releasing factor, thus increasing cohesin enrichments on chromatin, results in a significant increase of TAD size (Gassler et al., 2017; Haarhuis et al., 2017; Wutz et al., 2017). Conversely, deletion of NIPBL, a cohesin loading factor, or deletion of cohesin directly, causes weakening or loss of chromatin loops (Rao et al., 2017; Schwarzer et al., 2017).

### Asymmetric reeling of chromatin fibers by cohesin ‘loop extrusion’

In the *Pcdh* gene clusters, a large array of tandem forward CTCF sites in the variable region is followed by tandem reverse CTCF sites in the downstream super-enhancer (Guo et al., 2012; Zhai et al., 2016). CTCF/cohesin-dependent long-distance chromatin interactions bridge the distal enhancer to its target promoters and activate transcription. The reverse CTCF sites in the downstream super-enhancer act as a strong anchor to stall ‘one-head’ of cohesin complex. The other cohesin head still slides along the variable region and thus reels in chromatin fibers (Figure 6B). By inverting or deleting single or arrays of CTCF sites in the variable-promoter or super-enhancer regions of the clustered *Pcdh* genes and assaying the resulting architectural and functional consequences, asymmetric topological effects of long-distance chromatin contacts and disruption of *Pcdh* gene expression can be detected (Lu et al., 2019; Jia et al., 2020).

### Topological selections of enhancer‒promoter pairing

Genome-editing technologies have facilitated our understanding of 3D chromatin architecture in specific enhancer‒promoter contacts (reviewed in Schoenfelder and Fraser, 2019). CTCF/cohesin-mediated chromatin looping regulates the promoter selection of the *Pcdh* gene clusters and their neuron-specific expression patterns (Guo et al., 2012; Jiang et al., 2017; Allahyar et al., 2018; Wu et al., 2020). Specifically, the chromatin conformation capture 3C assay revealed that the enhancer element is spatially close to the promoter of the variable exon in the *Pcdh* gene cluster. In addition, the CTCF protein recognizes its conserved DNA-binding sites with directionality (Guo et al., 2015; Yin et al., 2017; Xu et al., 2018). Finally, single CTCF sites function as traditional insulators to ensure proper activation of target promoters by cognate enhancers; while tandem CTCF sites function as topological insulators to balance spatial chromatin contacts and to allocate enhancer resources for promoter choice (Zhai et al., 2016; Jia et al., 2020; Wu et al., 2020).

### Synthetic single-chromosome yeast

Double cutting by Cas9 guided by two sgRNAs, each targeting to a site close to the telomeres of two separate yeast chromosomes, leads to the fusion of the two chromosomes (Shao et al., 2018). Remarkably, a functional single-chromosome yeast was created by successive repeated fusions of all 16 yeast chromosomes into one giant chromosome by this CRISPR double cutting method (Shao et al., 2018). The two ends of the single linear chromosome could be further fused to generate a single circular chromosome (Shao et al., 2019). Apparently, both linear and circular single-chromosome yeasts have not been found in nature and thus are artificially synthesized yeast strains. This interesting observation indicates the power of targeted 3D genome engineering in synthetic biology by CRISPR with dual sgRNAs (Sadhu and Kruglyak, 2018).

### 3D genome synthetic biology

Programmed chromosomal fission and fusion by multiplexed CRISPR have generated synthetic genomes with nucleotide precision in bacteria (Wang et al., 2019a). In prokaryotic *Escherichia* *coli*, artificial chromosomes in single cells can be fused into a single genome with precise translocation and scarless inversion (Wang et al., 2019a). In eukaryotic yeast, Hi-C experiments revealed that the large-scale 3D organization of the synthetic genome is unaffected by the removal of numerous repeated sequences (Mercy et al., 2017). Interestingly, Hi-C experiments demonstrated that the single linear-chromosome and circular-chromosome yeasts have similar globular 3D genome conformation (Shao et al., 2019). These studies suggest that global 3D genome structures have significant plasticity and can tolerate local genetic perturbations.

## Perspective

We have sampled flavored highlights of some recent advances of genetic engineering of 3D genomes by CRISPR/Cas9 systems with various precise chromosomal rearrangements. Significant progress has been made recently in understanding the cleavage mechanisms of the CRISPR/Cas9 genome-editing system (Chen and Doudna, 2017). In addition, rapid technological advances in predictable DSB repair outcomes of precise CRISPR DNA fragment editing may accelerate its applications in agriculture and biomedicine (Tang and Fu, 2018). Furthermore, recent multiplexing CRISPR epigenetic technologies inform and promise cross-disciplinary revolutions (McCarty et al., 2020). Finally, CRISPR off-targets remain a big challenge but detecting methods are improving rapidly (Wienert et al., 2019).

Genetic engineering of 3D genomes and predictable chromosomal rearrangements by DNA fragment editing require interdisciplinary research. Obviously, fully predictable 3D genome engineering has not been achieved despite rapid progress in precise CRISPR DNA fragment editing in the last few years. Because very little is known in this area, it is a typical genre of desert-wandering night science that is full of darkness but also may stumble into a gold mine if lucky. 3D genomics integrates live biology with physical geometry. Renaissance of understanding and designing 3D genomes in the future may turn this night science into hypothesis-driven day science. Understanding the mechanisms of 3D genome folding will facilitate future precise and predictable CRISPR DNA fragment editing.
